# Functional transitions of the *Aspergillus fumigatus* iron regulator HapX are governed by conserved domains cooperatively binding [2Fe-2S] clusters

**DOI:** 10.1093/nar/gkaf796

**Published:** 2025-08-27

**Authors:** Simon Oberegger, Matthias Misslinger, Isidor Happacher, Hubertus Haas

**Affiliations:** Institute of Molecular Biology, Biocenter, Medical University Innsbruck, 6020 Innsbruck, Austria; Institute of Molecular Biology, Biocenter, Medical University Innsbruck, 6020 Innsbruck, Austria; Institute of Molecular Biology, Biocenter, Medical University Innsbruck, 6020 Innsbruck, Austria; Institute of Molecular Biology, Biocenter, Medical University Innsbruck, 6020 Innsbruck, Austria

## Abstract

Accurate sensing of cellular iron levels is vital, as this metal is essential but toxic in excess. The iron-sensing transcription factor HapX is crucial for virulence of *Aspergillus fumigatus*, the predominant human mold pathogen. Its absence impairs growth under iron limitation and excess, but not under moderate iron availability, suggesting that HapX switches between three states to adapt to varying iron availability. This study suggests that the HapX state transitions are regulated by different propensities of four phylogenetically conserved cysteine-rich regions (CRRs) to coordinate [2Fe-2S] clusters, resulting in cumulative occupancies depending on iron availability. The iron starvation state features no [2Fe-2S] clusters in any of the CRRs, the iron sufficiency/“neutral” state features clusters in CRR-B and/or -C, and the iron excess state has clusters in CRR-A, -B, and -C, while CRR-D plays a minor role. Combinatorial mutation of CRR-B and -C inhibited growth by locking HapX in the iron starvation state, leading to uncontrolled iron uptake and repression of iron-consuming pathways and iron detoxification. This growth defect was partially rescued by removing the C-terminal 27 amino acids, which are crucial for the iron starvation state and contain a degron. Noteworthy, the HapX iron starvation state induced several gene clusters encoding secondary metabolites.

## Graphical Abstract

**Graphical Abstract:**
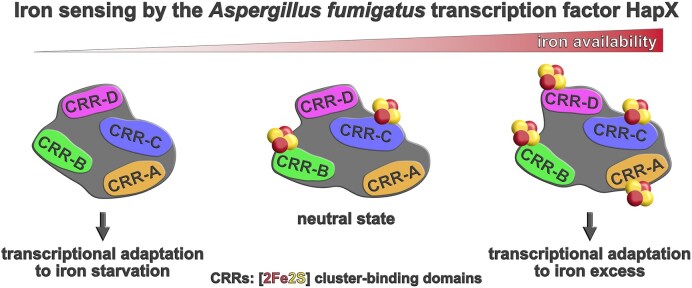

## Introduction

Iron is an indispensable trace element that is required by all eukaryotic and virtually all prokaryotic species [1]. The ability of iron to switch between two oxidation states, ferrous iron (Fe^2+^) and ferric iron (Fe^3+^), has made it an essential cofactor in redox biochemistry as heme, siroheme, iron–sulfur clusters, and mono- or dinuclear iron centers [2–5]. Iron-dependent pathways include respiration, the TCA cycle, oxidative stress detoxification, P450 enzymes, and DNA repair and replication, as well as the biosynthesis of amino acids, nucleotides, and sterols. Moreover, iron is also critically involved in less conserved pathways such as secondary metabolism [4]. Although iron is essential, excess iron leads to the formation of highly reactive hydroxyl radicals through Haber–Weiss–Fenton chemistry, causing oxidative cell damage [6, 7]. Therefore, sophisticated mechanisms are required to balance iron uptake, consumption, and storage to avoid both deficiency and toxicity of this metal, i.e. organisms must be able to discriminate cellular iron limitation (−Fe), iron sufficiency (+Fe), and iron excess (hFe) for respective adaptation.

*Aspergillus fumigatus* is one of the most common opportunistic fungal pathogens causing life-threatening invasive infectious diseases, particularly in immunocompromised patients, known as invasive aspergillosis [8, 9]. Iron homeostasis has been shown to be key to the virulence of this mold in various infection models [4]. In *A. fumigatus* iron homeostasis is mainly regulated by two iron-responsive transcription factors, termed HapX and SreA, which are interconnected in a negative transcriptional feedback loop [4]. In −Fe, HapX represses iron-consuming pathways, including heme biosynthesis, respiration, and vacuolar iron detoxification, while inducing high-affinity iron uptake through siderophore-mediated and reductive iron assimilation [10–12]. hFe turns HapX into an activator of iron-consuming pathways and vacuolar iron deposition by the transporter CccA [13, 14]. During sufficient iron supply, SreA represses expression of HapX and high-affinity iron acquisition by siderophore-mediated and reductive iron assimilation [15, 16]. Consequently, loss of HapX impairs growth in −Fe and hFe but not +Fe conditions, whereas loss of SreA impairs growth in +Fe and especially in hFe but not in −Fe conditions [10, 14, 15, 17].

SreA, a GATA-type zinc finger transcription factor, contains a single cysteine-rich region (CRR) for iron sensing [15, 18–20]. HapX shows several, evolutionarily highly conserved characteristics: (i) a Hap4-like domain mediating direct interaction with the CCAAT-binding complex for cooperative DNA binding [12, 21, 22], (ii) the bZIP-type DNA-binding domain, (iii) a coiled-coil domain for dimerization, (iv) four CRRs, termed CRR-A to CRR-D, that are involved in iron sensing, and (v) the C-terminal 27 amino acid region required for the role of HapX in −Fe but not hFe [4, 14, 21]. Each CRR of SreA and HapX contains four cysteine residues, which differ in spacing and amino acid composition of the spacer regions, and show high phylogenetic conservation [4, 23]. Several lines of evidence indicate that regulation of iron homeostasis via SreA and HapX depends on [2Fe-2S] cluster sensing by the CRRs: (i) inactivation of mitochondrial [2Fe-2S], but not cytosolic [4Fe-4S] cluster biogenesis, impaired iron sensing in *A. fumigatus* [24]; (ii) mutational analysis revealed that mainly CRR-B, but to a lesser extent also CRR-A and even less CRR-C, are required for HapX’s role in hFe but not −Fe, while mutation of CRR-D was phenotypically inconspicuous [14]; (iii) GrxD, which belongs to the [2Fe-2S] cluster-transferring monothiol glutaredoxins [25], physically interacts with both HapX and SreA and appears to mediate the removal of [2Fe-2S] clusters from both HapX and SreA to switch HapX from the “+Fe” to the “−Fe” state and to inactivate SreA [17]; (iv) the C-terminal domain of HapX (amino acids 161–491) containing the four CRRs, recombinantly expressed in *Escherichia coli*, exhibits a UV-vis spectrum indicative of [2Fe-2S] clusters [17]; and (v) peptide mimics of the CRRs of HapX and SreA have the ability to coordinate [2Fe-2S] clusters *in vitro* with differing propensities, whereby the [2Fe-2S] cluster coordinated by HapX CRR-B showed an outstanding high stability [23]. HapX and SreA are highly conserved in the fungal kingdom but not in Saccharomycetaceae, which employ different iron-sensing transcription factors [17]. Remarkably, the GrxD homologous monothiol glutaredoxins Grx3 and Grx4 are required for adaptation to iron sufficiency in *Saccharomyces cerevisiae* in contrast to the situation in *A. fumigatus*, where GrxD is important for −Fe adaptation [17, 26, 27]. These data highlight significant differences between iron homeostatic systems, even within fungal species.

Genetic inactivation of HapX causes growth and metabolic defects under −Fe and hFe but not +Fe [10, 14], indicating that this regulator can switch between three distinct states, mediating adaptation to −Fe, +Fe (neutral/inactive), and hFe. Similar to siderophore biosynthesis and uptake [28–31], HapX is crucial for *A. fumigatus* virulence in a murine model of aspergillosis and for *Fusarium oxysporum* in plant hosts, highlighting −Fe in the host niche and the importance of pathogen adaptation [10]. The latter demonstrates that switching HapX into its −Fe state is important for adaptation to the mammalian host niche. However, the molecular determinants of the HapX −Fe state have remained elusive. In this study, we elucidated the molecular mechanism for the HapX transitions between its three distinct functional states for adaptation to −Fe, +Fe (neutral/inactive), and hFe.

## Materials and methods

### Growth conditions

*Aspergillus* minimal medium [32] containing 1% (w/v) glucose and 20 mM glutamine as carbon and nitrogen sources, respectively, supplemented with iron-free trace elements was used to grow *A. fumigatus* strains. For phenotyping under different iron availabilities on solid media, media were supplemented with 0.2 mM bathophenanthrolinedisulfonic acid (BPS) without iron addition to limit bioavailability and to inhibit reductive iron assimilation [33] for −Fe conditions, 0.03 mM FeSO_4_ to reflect +Fe, or 10 mM FeSO_4_ to mimic hFe. For solid media, 1.8% (w/v) agar was added, and 10^4^ spores were point-inoculated. For liquid cultures, 100 mL minimal medium in 500 mL Erlenmeyer flasks was inoculated with 10^8^ spores and shaken at 200 revolutions per minute. BPS was not added in −Fe liquid cultures. For induction of the conditional *xylP* promoter (*pxylP*), xylose was added to a final concentration of 0.1% (w/v) [34, 35]. Cultures were generally incubated at 37 °C.

### Mutant strain generation

In this study, the non-homologous end joining-lacking (*ΔakuA::loxP*) ATCC46645 derivative AfS77, here referred to as wildtype (WT), was used [36]. Nucleic acid fragments for generation of plasmids were purified either by column purification or by gel extraction (Monarch PCR & DNA Cleanup Kit and Monarch DNA Gel Extraction Kit, New England Biolabs). Fragments were assembled using the NEBuilder HiFi DNA Assembly or applying site-directed mutagenesis (New England Biolabs); resulting plasmids were amplified in *E. coli* 5-alpha and purified using the Monarch Plasmid Miniprep Kit (New England Biolabs).

For generation of a strain expressing *pxylP*-driven *hapX* (strain *hapX*), the 1 kb *hapX* 5′-non-coding region for *hapX* locus-specific targeting was amplified from WT genomic DNA using primers *5′hapX flank_fwd*/*5′hapX flank_rev*, the *hph*-*pxylP* cassette was amplified from genomic DNA of strain *hapX^OE^* [37] using primers *hph-xylP cassette_fwd*/*hph-xylP cassette_rev*, and the *hapX* gene (Afu5g03920), including its native 1 kb 3′-non-coding region, was amplified from WT genomic DNA using primers *hapX-3′flank_fwd*/*hapX-3′flank_rev*. These fragments were assembled with the backbone of the commercially available plasmid pJet1.2 (Thermo Fisher Scientific) amplified using primers *pJet1.2 backbone_fwd*/*pJet1.2 backbone_rev* applying the NEBuilder approach, yielding plasmid pSO11. The *hph*-*pxylP* cassette contains the hygromycin resistance cassette [38], *hph*, and the *pxylP* promoter [34], which allows conditional expression of the *hapX* alleles.

The plasmid for the *hapX* version with combinatorial mutations of all four CRRs was based on plasmid pSO11 and was generated by site-directed mutagenesis. Therefore, a single cysteine residue of each CRR was mutated to an alanine residue (C203A, C277A, C353A, C380A) using mutagenesis primers, i.e. *oAfHapX-A2.f/oAfHapX-A2.r*, *oAfHapX-B1.f/oAfHapX-B1.r*, *oAfHapX-C3.f/oAfHapX-C3.r*, and *oAfHapX-D2.f/oAfHapX-D2.r*. The resulting plasmid pSO03, encoding the *hapX* allele designated *hapX^A/B/C/D^* (mutated CRRs are indicated in superscript letters), and plasmid pSO11 were subsequently used as templates to generate plasmids encoding the different combinations of CRR mutations; where appropriate, previous cysteine-to-alanine mutations were reversed to the required combinations of CRR mutations. The construction of the other plasmids is not described separately as they were constructed using the site-directed mutagenesis strategy as described for pSO03. C-terminal truncations of *hapX* and *hapX^A/B/C/D^*, resulting in *hapX^464^* and *hapX^A/B/C/D;464^*, were also generated using the site directed mutagenesis strategy using primers *oAfhapX-SO7/oAfhapX-SO8*. For fungal transformation, the transformation cassettes were polymerase chain reaction (PCR)-amplified from respective plasmids using primers *oAfhapX-1*/*oAfhapX-2*. After fungal transformation [39], transformants were selected using 100 μg/mL hygromycin.

For the generation of strains for *pxylP*-driven expression of Venus and a fusion protein of Venus and the C-terminal part of HapX (Venus^C-termHapX^), plasmid pSO52 was generated. Therefore, the following fragments were PCR amplified and assembled using the NEBuilder: (i) the plasmid backbone containing the *fcyB* 5′- and 3′-non-coding regions, the *pxylP*, and the *trpC* terminator derived from plasmid pSO16 [40] using primers *fcyB-bb_trpC_fwd/fcyB-bb_xylP_rev*; (ii) the Venus coding sequence derived from plasmid pSO16 [40] with the addition of a short linker sequence using primers *Venus_fwd/ Venus_linker-seq_rev*; and (iii) the C-terminal *hapX* coding region from WT gDNA using primers *linker-seq_hapX_fwd/hapX_rev*. Plasmid, pSO52 was then used as a PCR template to generate plasmids pSO53 and pSO54. Plasmid pSO53, encoding *Venus* only, was generated by site-directed mutagenesis using primers *pSO53_fwd/pSO53_rev*, deleting the *hapX* coding sequence. Plasmid pSO54, encoding *Venus* fused with the C-terminal 27 amino acid region of HapX, was generated by site-directed mutagenesis using primers *pSO54_fwd/pSO54_rev*. For fungal transformation, the plasmids were linearized by *Not*I restriction digestion and purified by column purification. In this case, no additional selection marker was required as locus-specific integration at the *fcyB* locus was used [41]. Therefore, transformants were selected using 10 μg/mL 5-fluorocytosine.

To generate strains overexpressing *cccA* under the control of the constitutive promoter *gpdA* (*cccA*^OE^; *pxylP:hapX* and *cccA*^OE^; *pxylP:hapX^A/B/C/D^*), the coding sequence of *cccA* was amplified from WT genomic DNA using the primers *cccA_fwd*/*cccA_rev*, the *gpdA* promoter was amplified from the pSO11 plasmid using the primers *gpdA promoter_fwd*/*gpdA promoter_rev*, and the *fcyB* targeting plasmid backbone including the *trpC* terminator was amplified from pSO16 [40] using the primers *fcyB-bb_trpC_fwd*/*fcyB-bb_rev*. These fragments were assembled using the NEBuilder approach, resulting in plasmid pSO51. As described earlier, plasmid linearization, fungal transformation, and selection of transformants were performed.

The generated strains, plasmids, and primers used in this study are summarized in Supplementary Tables S2–S4, respectively. All generated mutant strains were confirmed by Southern blot analysis; the results of the Southern blot analyses and schematic illustrations of the generated genotypes are shown in Supplementary Fig. S1.

### Isolation of RNA and DNA; Southern and northern blot analysis

Genomic DNA was isolated by PCI extraction and isopropanol precipitation. For Southern blot analysis, DNA digested with specified restriction enzymes was separated on a 0.7% (w/v) agarose gel and blotted on a Hybond^TM^-N membrane (Cytiva Amersham^TM^) with NaOH for denaturation.

Total RNA was extracted from freshly harvested biomass using TRI reagent (Sigma–Aldrich) according to the manufacturer’s protocol. For RNA gel electrophoresis, 10 μg of isolated RNA were loaded onto 1.2% (w/v) agarose gels with 1.85% (w/v) formaldehyde. For northern blot analysis, Hybond^TM^-N + membranes (Cytiva Amersham^TM^) were used.

Hybridization probes for Southern and northern blot analyses were generated by PCR amplification using digoxigenin-labeled deoxyribonucleotides (Roche); used primers are listed in Supplementary Tables S4 and S5, respectively.

### Transcriptome analysis using RNA sequencing

Illumina RNA library preparation, miCORE mRNA sequencing, and bioinformatic analysis were performed by Microsynth AG. Illumina reads were mapped to the reference genome, *A. fumigatus* Af293 ASM265 (NCBI RefSeq assembly: GCF_000002655.1), downloaded from NCBI database [42] using STAR version 2.7.11b [43]. The gene counts were normalized applying DESeq2 [44]. Differentially expressed genes were filtered using log_2_ fold change ≤ −1 or ≥ 1 and adjusted *P*-value < 0.05 as cut-offs. Visualization of the differential gene expression patterns in heat maps was performed using GraphPad Prism version 10.2.3 (GraphPad Software, www.graphpad.com). For functional categorization of the differentially expressed genes, the web-based tool FungiFun (version 2.2.8 BETA) was used [45].

### Determination of the cellular ferricrocin content and the chelatable iron pool

Cellular contents of iron-loaded ferricrocin were determined as described previously [24, 46]. Eighty milligrams of lyophilized mycelium was homogenized and resuspended in 1.8 mL sodium-phosphate buffer [pH 7.5; 50 mM] and incubated for 30 min on ice. Cellular debris was removed by centrifugation and 0.8 mL of the supernatant was transferred to fresh tubes and mixed with 0.2 mL of ROTI^®^ phenol/chloroform/isoamyl alcohol (50:24:1; Roth). After phase separation by centrifugation, 0.1 mL of the organic phase was transferred to fresh tubes and mixed with 0.1 mL water and 0.5 mL diethyl ether. After vigorous mixing and centrifugation, the ferricrocin concentration of the lower aqueous phase was measured at a wavelength of 434 nm using a nano spectrophotometer (Thermo Fisher Scientific).

The chelatable iron pool was quantified in the ferricrocin-depleted supernatant after ROTI^®^ phenol/chloroform/isoamyl alcohol (50:24:1; Roth) extraction and addition of BPS and ascorbic acid to final concentrations of 1 mM each, as previously described [24]. BPS-complexed ferrous iron was recorded at a wavelength of 535 nm using a molar extinction coefficient of *e* = 17 000 L mol^−1^ cm^−1^.

### Protein extraction and western blot analysis

Total protein was extracted from lyophilized biomass (Triad freeze dryer, Labconco) by alkaline lysis, in combination with trichloroacetic acid-mediated protein precipitation, as reported previously [47]. Total protein extracts were separated electrophoretically on 12% (w/v) sodium dodecyl sulfate (SDS)–polyacrylamide gels and transferred onto nitrocellulose membranes (Amersham^TM^ Protran^TM^ Premium 0.45 μm NC, Cytiva Amersham^TM^) using the Trans-Blot^®^ Turbo™ System (Bio-Rad Laboratories).

HapX proteins were recorded using a rabbit α-HapX antibody [17], 1:10 000 diluted, in combination with a secondary, peroxidase-coupled α-rabbit antibody (Sigma–Aldrich), 1:20 000 diluted, using ECL detection (Cytiva Amersham^TM^). Protein amounts were estimated densitometrically using Fiji ImageJ [48].

Venus proteins were detected using a mouse α-GFP antibody (1:10 000 diluted; Roche) as the primary antibody in combination with a peroxidase-coupled secondary anti-mouse antibody (1:10 000 diluted; Sigma–Aldrich). The ECL reagent (Cytiva Amersham^TM^) was used for detection.

### Bioinformatics

Required gene and protein sequences were downloaded from FungiDB [49] or from the NCBI database [42]. Plasmid and protein sequences were generated and maintained using the cloud-based platform Benchling (Biology Software, 2023). Sequence alignments of proteins were generated using Geneious Prime (2024.0.5; https://www.geneious.com) [50]. The predicted protein structure of *A. fumigatus* HapX was obtained from AlphaFold (AF-Q4WER3-F1-v4) [51, 52]. Visualization was done using PyMOL Molecular Graphics System, Version 3.0.3, Schrödinger, LLC [53].

### Statistical analyses

Statistical analyses were carried out applying GraphPad Prism version 10.2.3 (GraphPad, www.graphpad.com).

## Results

### Combinatorial mutation of HapX CRR-A, CRR-B, CRR-C, and CRR-D blocks growth

**Figure 1.**
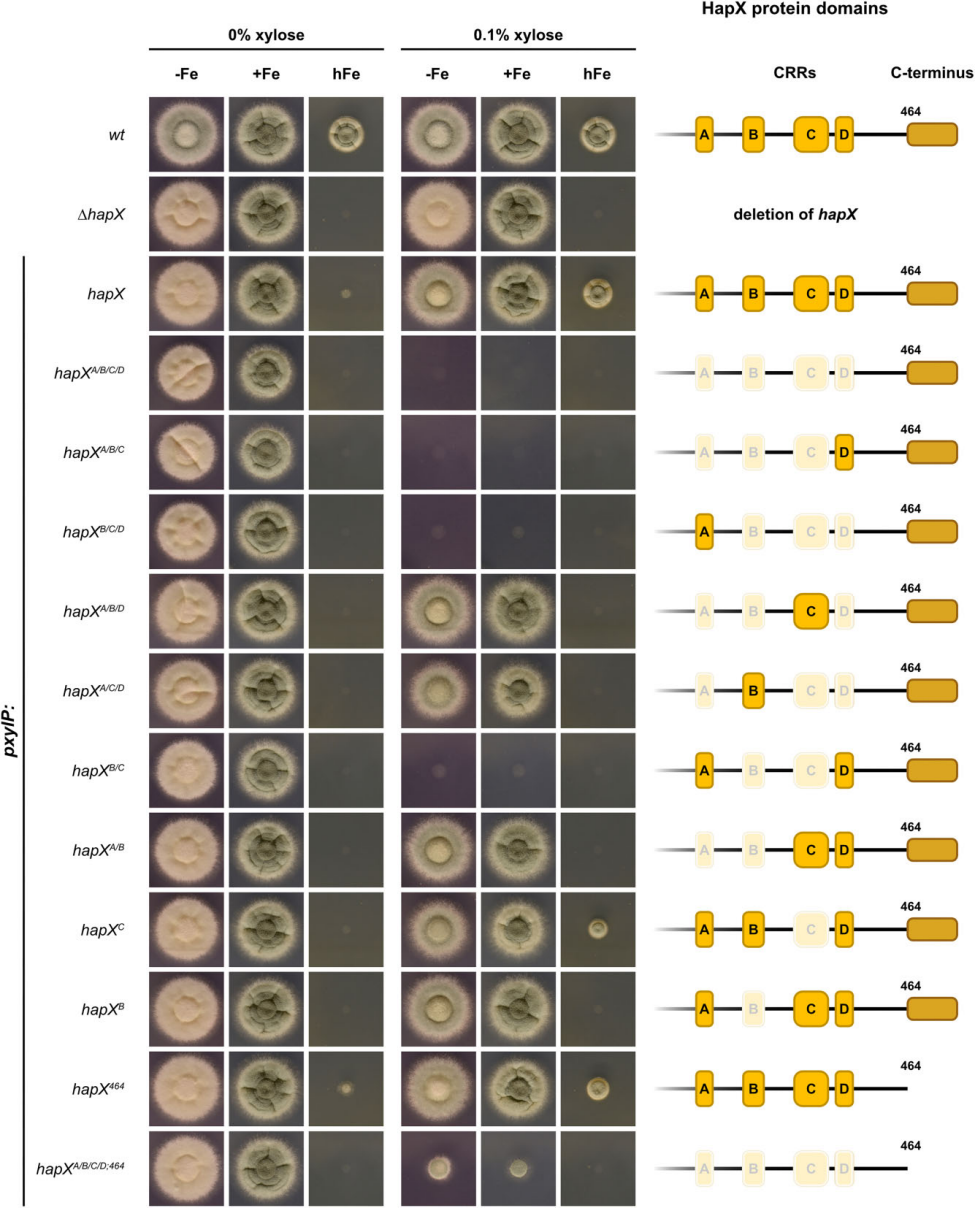
Combinatorial mutation of CRR-B and -C (strains *hapX^A/B/C/D^*, *hapX^A/B/C^*, *hapX^B/C/D^*, *hapX^B/C^*), but not their individual mutation (*hapX^B^*, *hapX^C^*), blocks growth independent of iron availability (−Fe / +Fe / hFe). For each fungal strain, 10^4^ conidia were spot-inoculated on minimal medium plates reflecting iron starvation (−Fe; 200 μM BPS), iron sufficiency (+Fe; 30 μM FeSO_4_) or iron excess (hFe; 10 mM FeSO_4_). Expression of *pxylP*-driven *hapX* alleles was tuned by supplementation with xylose: 0% for repression and 0.1% for moderate expression. Plates were incubated at 37 °C for 48 h. The CRRs and the C-terminus of the respective HapX alleles are schematically shown on the ride side; functional inactivation of a CRR is indicated by white shading.

Previously, mutational analysis in HapX revealed that mainly CRR-B, but to a lesser extent also CRR-A and even less CRR-C, are required for HapX’s role in hFe but not −Fe, while mutation of CRR-D was phenotypically inconspicuous [14]. Important to note, individual replacement of different cysteine residues of the same CRR by alanine had the same phenotypical consequences, indicating that the four cysteine residues in a CRR function in concert and that replacement of a single cysteine residue is sufficient to inactivate the function of a CRR [14]. Attempts to generate a HapX allele expressed under its native promoter and lacking all four CRRs were unsuccessful, suggesting that this might be lethal. For further functional analysis of HapX protein domains, generated *hapX* alleles were therefore expressed under control of the xylose-inducible *xylP* promoter (*pxylP*), which allows tunable expression levels, including repression and overexpression [34, 35], with single copy integration at the native *hapX* locus. For phenotyping, fungal strains were grown on solid media reflecting −Fe, +Fe and hFe without xylose for repression and 0.1% xylose supplementation for expression of *pxylP*-driven *hapX* alleles (Fig. 1). Compared to +Fe, *A. fumigatus* WT displayed reduced conidiation (reduced green coloration of the fungal colony) in −Fe and reduced radial growth in hFe, largely independent of xylose supplementation. Compared to WT, lack of HapX (*ΔhapX*) decreased conidiation in −Fe and blocked growth in hFe but showed WT-like growth in +Fe, independent of xylose supplementation, as previously reported [14]. Without xylose supplementation, *pxylP*-driven native *hapX* displayed a *ΔhapX* growth phenotype, apart from slight growth in hFe most likely due to slight leakiness of *pxylP* [35]. With xylose induction, *pxylP*-driven native *hapX* displayed a largely WT-like growth pattern (apart from mildly decreased growth in hFe), demonstrating the suitability of *pxylP* for studying functionality of *hapX* alleles.

As a first step in the functional analysis of the HapX CRRs, we simultaneously replaced one cysteine of each CRR by an alanine residue (C203A, C277A, C353A, C380A) to block [2Fe-2S] cluster binding by all four CRRs, resulting in strain *hapX^A/B/C/D^*. Under non-inducing conditions (0% xylose), this strain showed a growth pattern similar to that of *ΔhapX* (Fig. 1). Since the latter growth behavior was observed for all generated mutant strains without xylose supplementation (Fig. 1), it is not discussed further for the other generated strains. Remarkably, xylose-induced expression of the *hapX^A/B/C/D^* allele blocked growth under all conditions tested (Fig. 1). Importantly, individual mutation of the four CRRs of HapX expressed under the endogenous promoter was previously shown to negatively affect growth only in hFe, whereby this was observed mainly for CRR-B, less for CRR-A, only mildly for CRR-C, and not at all for CRR-D [14]. Similarly, under *pxylP* control, individual mutation of CRR-B (strain *hapX^B^*) blocked, and that of CRR-C (strain *hapX^C^*) mildly decreased growth exclusively in hFe compared to the native *hapX* allele with xylose induction (Fig. 1).

**Figure 2.**
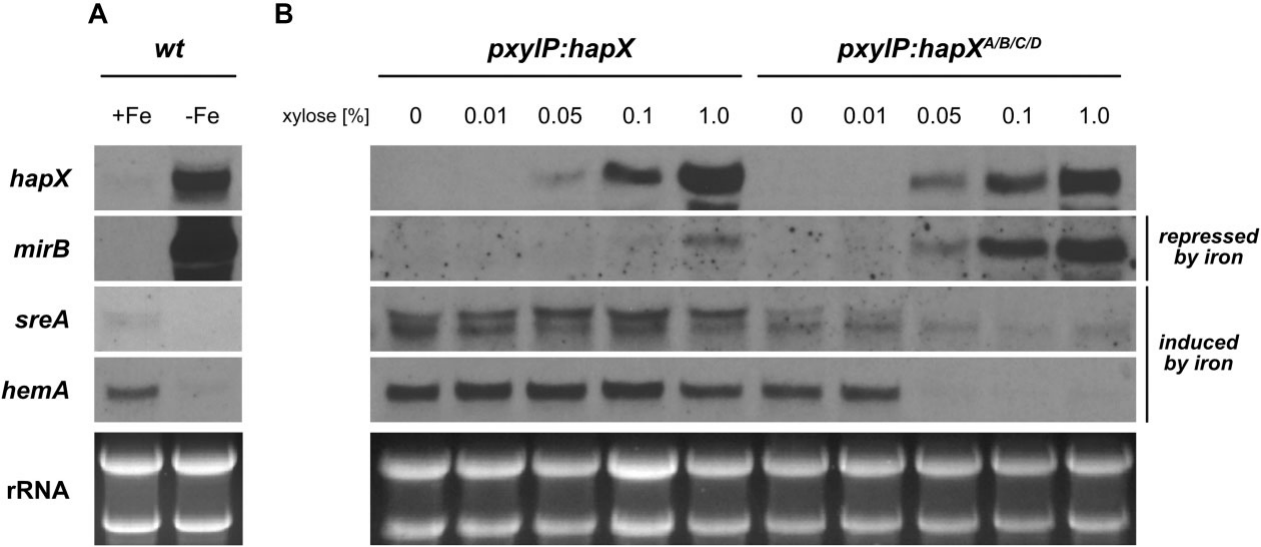
Combinatorial mutation of all four HapX CRRs (*hapX^A/B/C/D^*) results in an iron starvation response in +Fe. Total RNA was isolated from liquid cultures grown either in +Fe or −Fe at 37 °C. The WT (**A**) was grown for 18 h, strains expressing *pxylP*-driven *hapX* and *hapX^A/B/C/D^* (**B**) were grown in +Fe without xylose for 17 h followed by 1 h incubation with different xylose concentrations for induction of *hapX* expression. Northern blot analyzed genes *hapX*, iron limitation-induced *mirB*,and iron limitation-repressed *sreA* and *hemA*. Ethidium bromide-stained rRNA is shown for loading and quality control.

To investigate the reason for the growth-blocking effect of *hapX^A/B/C/D^* allele expression, we compared the expression of HapX target genes in strains expressing the *pxylP*-driven native *hapX* and *hapX^A/B/C/D^* alleles by northern blot analysis. Therefore, *hapX* and *hapX^A/B/C/D^* strains were grown in +Fe liquid cultures without xylose supplementation for 17 h to circumvent the growth inhibitory effect of combinatorial mutation of all four CRRs, taking advantage of the phenotypically inconspicuous *ΔhapX* phenotype in +Fe (Fig. 1), followed by incubation for 1 h in the presence of varying amounts of xylose to induce expression of the *hapX* alleles. Additionally, the WT was grown for 18 h in −Fe and +Fe liquid cultures as control. Harvested mycelia were subjected to northern blot analysis of iron-regulated genes. In line with previously reported data [10, 14], *hapX* transcript levels were strongly upregulated in −Fe compared to +Fe in WT (Fig. 2A). In contrast, strains with *pxylP*-driven *hapX* showed *hapX* expression that increased with xylose supplementation from 0.05 to 1.0% xylose, whereas *hapX* transcripts were not detected without (0%) or low (0.01%) xylose supplementation (Fig. 2B). In contrast to the *hapX* strain, with xylose induction ≥ 0.05%, the *hapX^A/B/C/D^* strain showed an expression pattern similar to that of the WT grown in −Fe (Fig. 2B), i.e. induction of −Fe-induced *mirB*, which mediates siderophore uptake [54], and repression of −Fe-repressed heme biosynthetic *hemA* and iron regulatory *sreA* (Fig. 2B). This expression pattern of strain *hapX^A/B/C/D^* is consistent with the previously reported −Fe response in *A. fumigatus* [10, 14, 15, 17]. These data strongly suggest that the simultaneous mutational disruption of all four HapX CRRs renders HapX iron-blind, regardless of the prevailing iron availability, as shown here for +Fe. As shown previously [15, 19], *sreA* expression resulted in two different-sized transcripts due to alternative transcription initiation. Notably, expression of *hapX^A/B/C/D^* mainly affected the larger *sreA* transcript (Fig. 2B), revealing differential transcriptional regulation of the two *sreA* transcripts by HapX. Remarkably, high expression (1.0% xylose) of the WT*hapX* allele resulted in weak induction of *mirB* (Fig. 2B). Most likely, short-term production of large amounts of HapX protein exceeds the transfer capacity for [2Fe-2S] clusters, and consequently a fraction of the cellular HapX pool is in the −Fe state even in +Fe. In contrast to the loss of HapX, which blocks growth exclusively in hFe, combinatorial mutation of all four CRRs blocked growth in all conditions. Apparently, the constitutive activation of iron acquisition combined with the constitutive repression of iron-consuming pathways and iron detoxification caused by the *hapX^A/B/C/D^* allele is more deleterious than the de-repression of iron-consuming pathways and reduction of iron acquisition in −Fe caused by loss of HapX.

### Combinatorial mutation of all four CRRs locks HapX in the −Fe status causing cellular stress and affecting secondary metabolism

**Figure 3.**
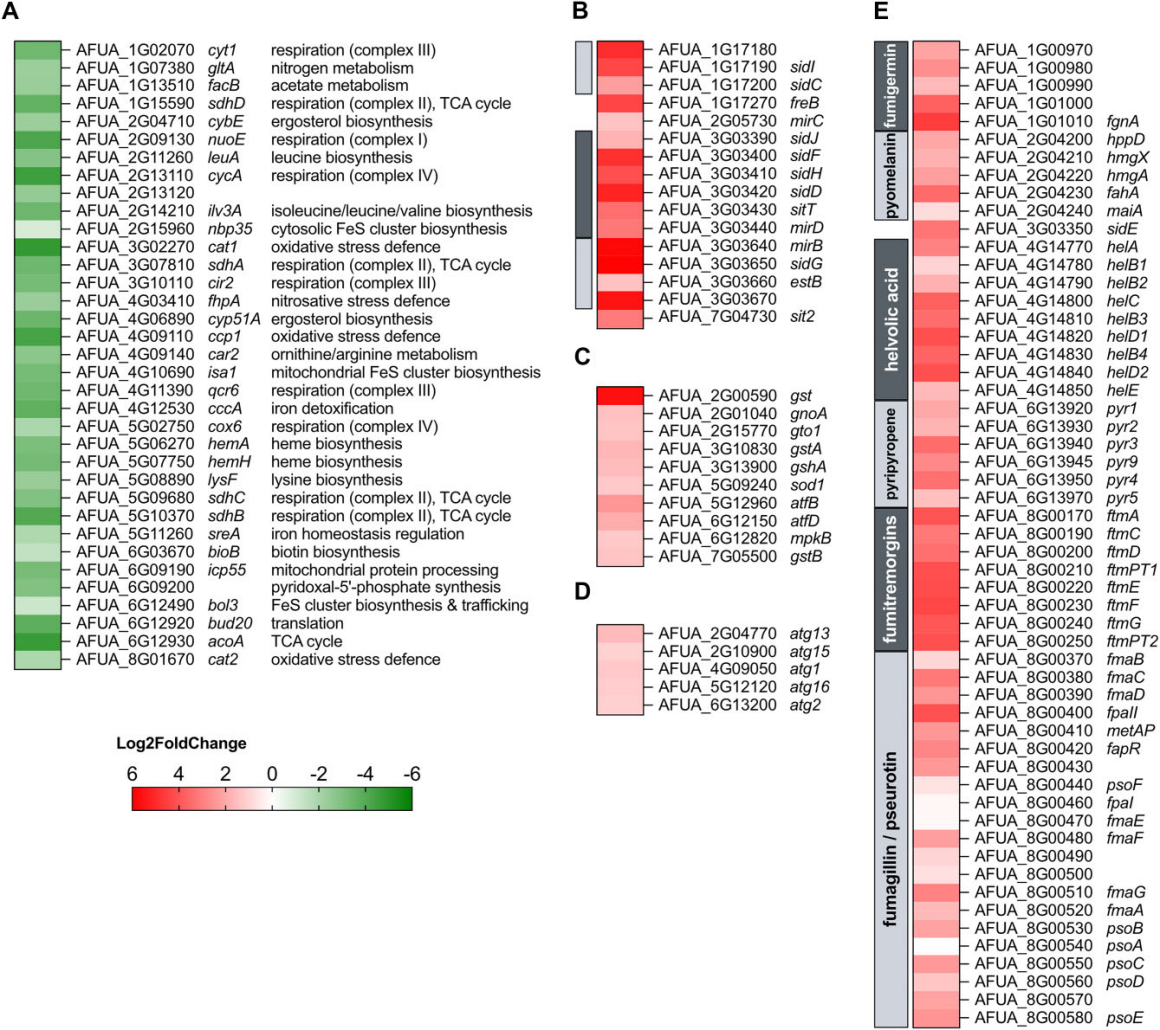
Heatmap comparison of selected differentially expressed genes in *hapX^A/B/C/D^* compared to *hapX* in+Fe. Genes that have previously been shown to be directly downregulated by HapX in −Fe (**A**); genes involved in siderophore metabolism or localized in siderophore metabolic gene clusters that have previously been shown to be upregulated in −Fe (**B**); genes involved in stress response (**C**); genes involved in autophagy (**D**); and genes involved in secondary metabolism (**E**). Only genes downregulated with a log_2_ fold change ≤ −1 or upregulated with ≥ 1 are shown, with the exception of (**E**): here, all genes of respective secondary metabolite encoding gene clusters are shown. Fumarylalanine is synthesized by SidE, most likely without the need for other enzymes. Gene clusters are highlighted by gray shaded boxes on the left side of the heat maps. The respective transcriptome data subsets are shown in Supplementary Table S1B.

The northern blot analysis of selected genes shown earlier (Fig. 2) cumulatively suggested that combinatorial mutation of the four CRRs locks HapX in the −Fe state. To characterize the genome-wide effect of combinatorial mutation of the four CRRs, we employed high-throughput RNA sequencing for transcriptome profiling. Therefore, biological triplicates of strains encoding *pxylP*-driven *hapX* and the *hapX^A/B/C/D^* were grown in liquid culture in +Fe for 17 h to allow growth under non-inducing conditions followed by an incubation for 1 h in the presence of 0.1% xylose for induction of the *hapX* alleles. On average, we obtained 23 476 866 reads per individual sample. Genome alignment revealed an average unique mapping percentage of 82.5% to the *A. fumigatus* Af293 reference genome, identifying the expression of 9859 genes, of which 1406 were found to be upregulated and 1175 were downregulated ≥ 2-fold in strain *hapX^A/B/C/D^* compared to *hapX* with an adjusted *P*-value < 0.05 (Fig. 3). The transcriptome data are shown in Supplementary Table S1A; principal component and volcano plot analyses of the transcriptomic comparison are shown in Supplementary Fig. S2.

A previous study identified 33 genes that are directly repressed by the CBC:HapX complex in −Fe [12]. These genes encode iron-linked steps in various pathways, including respiration, TCA cycle, CccA-mediated vacuolar iron deposition, defense against oxidative and nitrosative stress by catalase and flavohemoglobin, and SreA, as well as biosynthesis of heme, iron-sulfur clusters, and amino acids. All these genes were downregulated in *hapX^A/B/C/D^* compared to *hapX* (Fig. 3A). Moreover, the majority of genes involved in siderophore-mediated iron assimilation or localized in encoding gene clusters that have been previously shown to be repressed by SreA [15, 17] were upregulated in *hapX^A/B/C/D^* compared to *hapX* (Fig. 3B). Taken together, these results demonstrate that combinatorial mutation of the four CRRs locks HapX in the −Fe state, leading to a transcriptional −Fe response in +Fe conditions. Therefore, the most likely reason for the severe effect of combinatorial mutation of the four CRRs described earlier is uncontrolled iron acquisition combined with repression of iron-consuming pathways and CccA-mediated vacuolar iron detoxification.

Iron overload is expected to cause cellular stress, particularly oxidative and nitrosative stress due to the formation of highly reactive oxygen and nitrogen species, also known as Haber–Weiss–Fenton and Fenton-like chemistry [6, 7, 55, 56]. In line, several genes encoding stress-responsive regulatory or structural proteins were found to be upregulated in strain *hapX^A/B/C/D^* (Fig. 3C), including the mitogen-activated protein kinase MpkB [57], the basic leucine zipper (bZIP) transcription factors AtfB and AtfD [58], glutathione biosynthetic GshA [24], several glutathione transferases Gto1, GstA, and GstB [59], Cu/Zn-superoxide dismutase SodA [60], and nitrosative stress-detoxifying S-nitrosoglutathione reductase GnoA [61]. Noteworthy, the genes encoding oxidative stress-detoxifying mycelial catalases Cat1 and Cat2 [62] as well as nitrosative stress-detoxifying flavohemoglobin FhpA [61] were significantly downregulated in strain *hapX^A/B/C/D^* (Fig. 3A). However, these genes have previously been shown to be directly repressed by HapX during −Fe [12, 14]. Consequently, the activation of the −Fe state of HapX in +Fe appears to result not only in uncontrolled iron uptake combined with repression of iron consumption and detoxification but most likely also impairs defense against oxidative and nitrosative stress.

To analyze the role of iron in the growth defect caused by combinatorial mutation of the four CRRs, we constitutively expressed in this mutant *cccA*, which encodes a transporter that mediates iron detoxification by vacuolar iron deposition [13, 14] and is repressed by the *hapX^A/B/C/D^* allele (Fig. 3A), using the strong *gpdA* promoter [63, 64]. The observed slight rescue of the growth defect (Supplementary Fig. S3) indicates that repression of vacuolar iron deposition does indeed play a role in the growth defect, but is not the sole cause.

Remarkably, the majority of the downregulated genes in strain *hapX^A/B/C/D^* are related to ribosome biogenesis and translation (Supplementary Fig. S4 and Supplementary Table S1C), while five genes involved in autophagy were upregulated (Fig. 3D), a pattern consistent with repression of TOR complex 1 (TORC1; target of rapamycin) [65, 66].

In addition, biosynthetic gene clusters for the secondary metabolites fumigermin [67], pyomelanin/tyrosine degradation [68], fumarylalanine [69], helvolic acid [70], the meroterpenoid pyripyropene [71], fumitremorgins [72], and fumagillin/pseurotin [73, 74] were upregulated in strain *hapX^A/B/C/D^*.

Notably, induction of fumarylalanine and pyomelanin/tyrosine degradation has previously been linked to cellular stress [68, 69]. In contrast, expression of gene clusters [75] for biosynthesis of fumigaclavines (*fga*), hexadehydroastechrome (*has*), isocyanides fumivaline/fumicicolin (*crm*), endocrocin (*enc*), trypacidin (*tpc*), xanthozillin (*xan*), fumisoquin (*fmp/fsq*), gliotoxin (*gli*), neosartoricin/fumicycline (*nsc*), trypacidin (*tpc*), fumihopaside (*afum*), and satorypyrone (spy) was largely unaffected in strain *hapX^A/B/C/D^* compared to *hapX* (Supplementary Table S1A).

### Combinatorial mutation of HapX CRR-B and CRR-C blocks growth and activates the −Fe state

To elucidate the combinatorial mutation which is responsible for the growth inhibitory effect, i.e. which CRRs are involved in locking HapX in the −Fe state, we generated mutant strains expressing *hapX* alleles carrying all combinatorial mutations of three CRRs, termed *hapX^A/B/C^*, *hapX^B/C/D^*, *hapX^A/B/D^* and *hapX^A/C/D^*. Phenotyping revealed that only *hapX^A/B/C^* and *hapX^B/C/D^* strains phenocopied the *hapX^A/B/C/D^* strain upon xylose induction (Fig. 1), suggesting that the combinatorial mutation of CRR-B and -C, which is common to these two alleles, is most likely responsible for the growth defect. Indeed, combinatorial mutation of only CRR-B and -C (strain *hapX^B/C^*) blocked growth upon xylose induction, regardless of the prevailing iron availability (Fig. 1). In contrast, individual mutation of CRR-B (strain *hapX^B^*) or CRR-C (strain *hapX^C^*) or combinatorial mutation of CRR-A and -B (strain *hapX^A/B^*) impaired growth only in hFe (Fig. 1). Taken together, these results demonstrate that combinatorial mutation of CRR-B and -C is sufficient to block growth, regardless of the prevailing iron availability. As previously shown in strains expressing *hapX* under its native promoter [14], individual mutation of CRR-B (strains *hapX^B^, hapX^A/B^*, *hapX^A/B/D^*) blocked and mutation of CRR-C (strain *hapX^C^*) reduced growth exclusively in hFe (Fig. 1). Moreover, combinatorial mutation of CRR-A, -C, and -D (strain *hapX^A/C/D^*) blocked growth in hFe (Fig. 1). These results highlight the importance of CRR-A, -B, and -C in mediating iron resistance in *A. fumigatus*, as previously demonstrated with individual mutation of CRRs [14]. Mutation of CRR-D was phenotypically inconspicuous in all combinations tested (Fig. 1).

**Figure 4.**
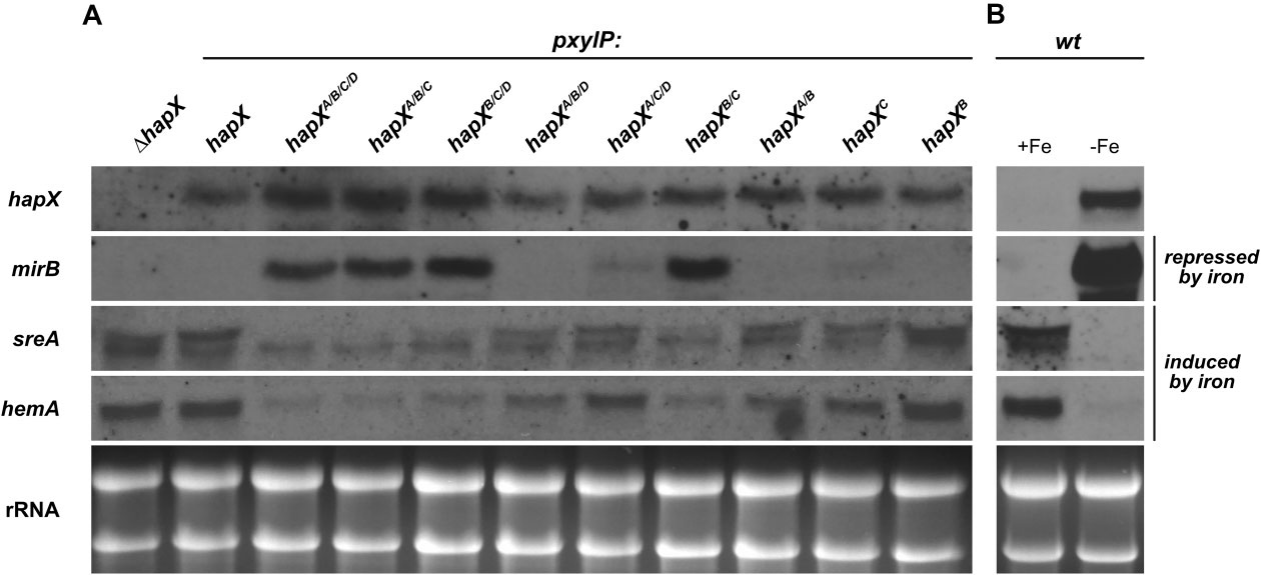
Combinatorial mutation of CRR-B and CRR-C (strains *hapX^A/B/C/D^*, *hapX^A/B/C^*, *hapX^B/C/D^* and *hapX^B/C^*) locks HapX in the iron limitation state, i.e. causes a transcriptional iron starvation response in +Fe. Total RNA was isolated from liquid cultures grown in +Fe (**A**) or +Fe and −Fe (**B**) at 37 °C. Strains were grown in +Fe without xylose for 17 h followed by 1 h incubation with 0.1% xylose for induction of *hapX* expression (A), and WT was grown for 18 h without xylose supplementation (B). Northern blot analyzed genes: *hapX*, iron limitation-induced *mirB*, and iron limitation-repressed *sreA* and *hemA*. Ethidium bromide-stained rRNA is shown for loading and quality control.

All generated strains expressing *hapX* alleles were subjected to northern blot analysis in +Fe with xylose induction for 1 h (Fig. 4). In contrast to *ΔhapX*, all strains expressing *pxylP*-driven *hapX* alleles showed largely similar *hapX* induction (Fig. 4A), whereby *hapX* transcript levels were slightly higher in strains *hapX^A/B/C/D^*, *hapX^A/B/C^*, and *hapX^B/C/D^* compared to the other strains. Similar to the strain expressing *hapX* with combinatorial mutation of CRR-A, -B, -C, and -D (*hapX^A/B/C/D^*), all other strains with combinatorial mutation of CRR-B and -C (strains *hapX^A/B/C^*, *hapX^B/C/D^*, and *hapX^B/C^*), in contrast to the remaining strains (*ΔhapX*,*hapX*,*hapX^A/B/D^*,*hapX^A/C/D^*,*hapX*^*A/B*^,*hapX*^*C*^ and *hapX^B^*), showed a transcriptional −Fe response in +Fe, i.e. higher transcript levels of *mirB* and lower transcript levels of *hemA* and the larger *sreA* transcript (Fig. 4A), similar to WT in −Fe (Fig. 4B). Again, mutation of CRR-D did not affect the transcriptional pattern in any combination. Taken together, these data underscore that the combinatorial mutation of CRR-B and -C blocks growth by causing “iron blindness” of HapX. In turn, these data suggest that CRR-B and -C do not coordinate [2Fe-2S] clusters in −Fe and that [2Fe-2S] cluster occupancy of CRR-B and/or -C switches HapX out of the iron starvation state.

### C-terminal HapX truncation partially rescues the growth defect caused by combinatorial mutation of the four CRRs

The C-terminal 27 amino acid residues of HapX (amino acid residues 465–491) have previously been shown to be crucial adaptation to −Fe but not hFe [14]. To investigate a possible link between the HapX C-terminus and the CRRs, we generated mutants expressing *pxylP*-driven *hapX* lacking the C-terminal 27 amino acid residues without (strain *hapX^464^*) or with combinatorial mutation of the four CRRs (*hapX^A/B/C/D;464^*). As previously shown with native promoter control [14], the C-terminal HapX truncation (strain *hapX^464^*) had no significant effect on growth in hFe (Fig. 1). Remarkably, the C-terminal truncation partially rescued the growth defect caused by mutation of the four CRRs with xylose induction in −Fe and +Fe but not in hFe (Fig. 1).

**Figure 5.**
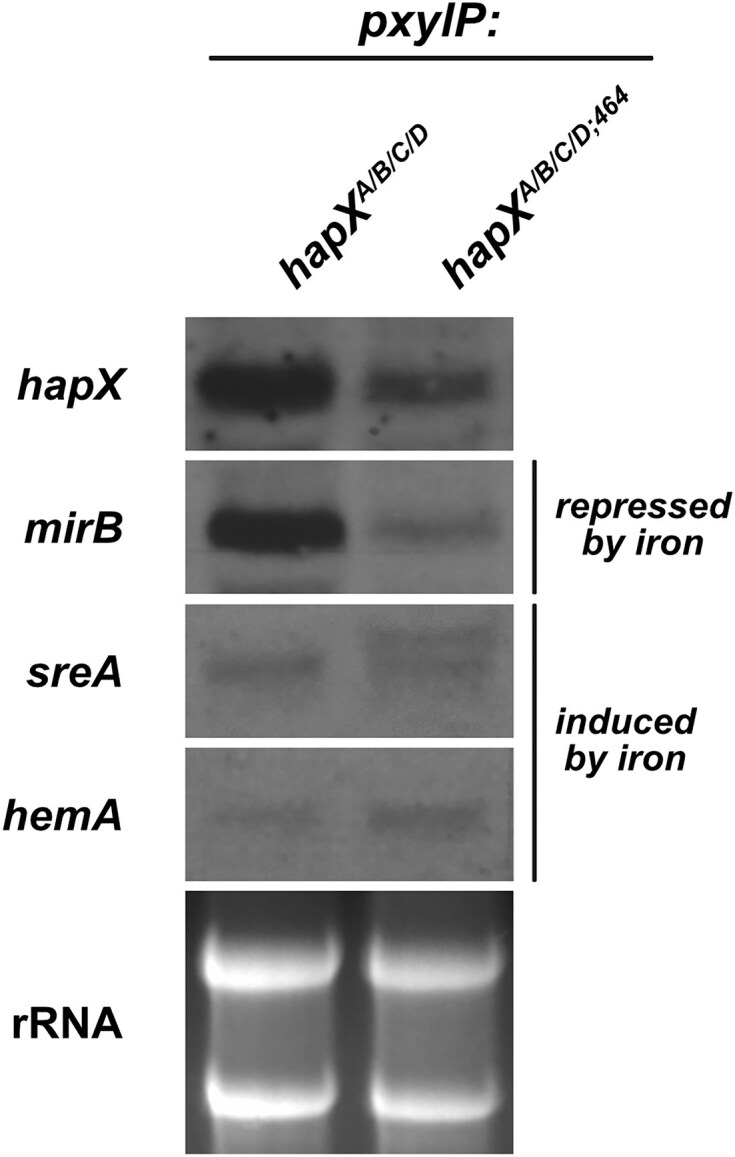
C-terminal HapX truncation (strain *hapX^A/B/C/D,^^464^*) diminishes the transcriptional iron starvation response in +Fe caused by combinatorial mutation of the four CRRs (strain *hapX^A/B/C/D^*). Total RNA was isolated from liquid cultures grown in +Fe at 37 °C for 17 h without xylose followed by 1 h incubation with 0.1% xylose for induction of *pxylP*-driven *hapX* alleles. Northern blot analyzed genes: *hapX*, iron limitation-induced *mirB*, and iron limitation-repressed *sreA* and *hemA*. Ethidium bromide-stained rRNA is shown for loading and quality control.

Northern blot analysis of mycelia grown in +Fe with xylose induction for 1 h revealed that truncation of the C-terminal 27 amino acid region (strain *hapX^A/B/C/D;464^*) attenuated the transcriptional iron starvation response caused by combinatorial mutation of the four CRRs (strain *hapX^A/B/C/D^*), i.e. it decreased the *mirB* transcript level and increased the level of the larger *sreA* transcript, while the *hemA* transcript level was not affected (Fig. 5).

Taken together, the fact that the truncation of the C-terminal 27 amino acid residues, which have previously been shown to be crucial for −Fe adaptation [14], partially rescued the growth defect and attenuated the transcriptional −Fe response caused by combinatorial mutation of the four CRRs underlines that the growth defect is caused by the severe −Fe response.

### Growth inhibition caused by combinatorial mutation of at least CRR-B and -C is more severe in +Fe compared to −Fe and accelerates with increased expression of the corresponding *hapX* alleles.

**Figure 6.**
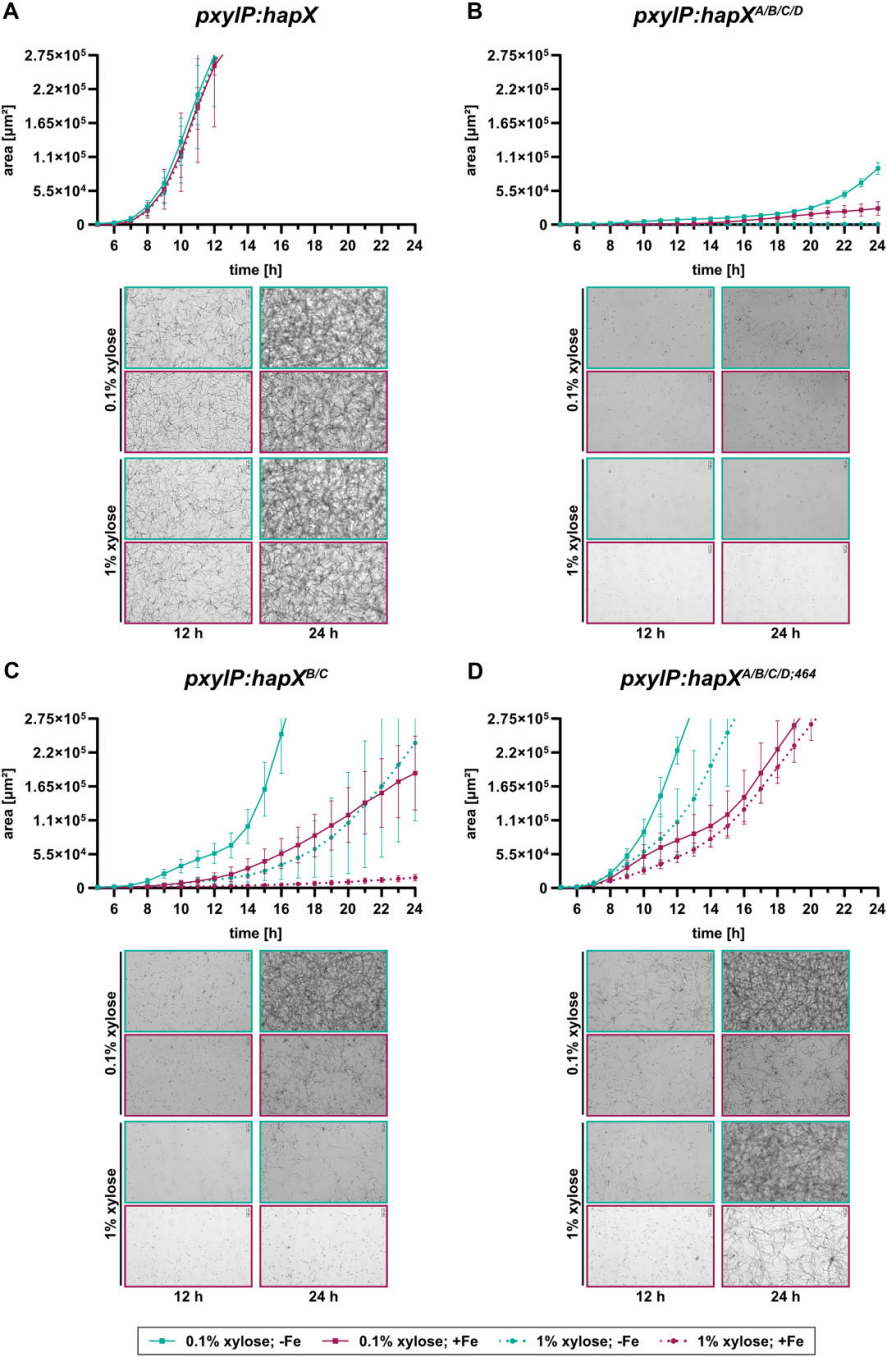
In liquid media, growth inhibition by combinatorial mutation of at least CRR-B and -C is more severe in +Fe compared to −Fe and accelerates with increased expression of the corresponding *hapX* alleles. For each strain, (**A**) *pxylP:hapX*, (**B**) *pxylP:hapX^A/B/C/D^*, (**C**) *pxylP:hapX^B/C^*, and (**D**) *pxylP:hapX^A/B/C/D,464^*, 10^4^ conidia were inoculated in triplicate into 96-well microplates in 0.1 mL minimal medium reflecting iron limitation (−Fe; in green) and iron sufficiency (+Fe; 10 μM FeSO_4_; in red) supplemented with 0.1% xylose for moderate and 1% xylose for high expression of the respective *hapX* allele. Using the oCelloScope (BioSense Solutions, Denmark) and its UniExplorer software (version 14.0), growth was scored hourly at 37 °C from 2 h post-inoculation (graphs start at the 5 h time point as there was very limited growth before that) until 24 h incubation. In the software, the “*fungi filamentous growth module*” was used to assess the hyphal area up to 2.75 × 10^5^ μm^2^ when the growth measurement reached saturation. The values in the growth curves represent the mean ± standard deviation of biological triplicates. Below the growth curves are examples of microscopic images of the fungal cultures taken automatically by the oCelloScope at two time points, 12 h and 24 h, underlining the scored growth data.

As an alternative to growth analysis on solid media (Fig. 1), the growth of strains expressing *pxylP*-driven *hapX*, *hapX^A/B/C/D^*, *hapX*^*B/C*^ and *hapX^A/B/C/D;464^* was analyzed in liquid media. Therefore, we applied automated microbial live cell imaging and analysis using the oCelloScope (BioSense Solutions ApS, Denmark) measuring growth by determining hyphal area (Fig. 6). The growth of the four strains was compared in −Fe and +Fe supplemented with either 0.1% xylose for moderate or 1% xylose for high expression of the respective *hapX* allele. Under these conditions, neither iron availability nor the *hapX* expression level affected the growth of the strain expressing the native *hapX* allele (Fig. 6A). In comparison, combinatorial mutation of the four CRRs (strain *hapX^A/B/C/D^*) significantly inhibited growth, with higher inhibition in +Fe compared to −Fe with 0.1% xylose supplementation and complete inhibition with 1% xylose supplementation regardless of iron availability (Fig. 6B). Combinatorial mutation of CRR-B and CRR-C (strain *hapX^B/C^*) resulted in decreased growth compared to the native *hapX* allele, but significantly increased growth compared to combinatorial mutation of the four CRRs (Fig. 6C). Growth of this strain was significantly enhanced in −Fe compared to +Fe and with 0.1% xylose compared to 1% xylose supplementation (Fig. 6C). Compared to combinatorial mutation of the four CRRs (strain *hapX^A/B/C/D^*) (Fig. 6B), truncation of the C-terminal 27 amino acid residues in this allele (*hapX^A/B/C/D;464^*) significantly increased the growth, as seen also on solid media (Fig. 1), again with enhanced growth in −Fe compared to +Fe and with 0.1% xylose compared to 1% xylose supplementation (Fig. 6D).

Taken together, these results demonstrate that *hapX* alleles with combinatorial mutation of at least CRR-B and CRR-C reduce growth compared to the native *hap*X allele, with greater growth inhibition in +Fe compared to −Fe, consistent with constitutive activation of the HapX −Fe state by these alleles (Figs 2 and 3). Furthermore, the data show that increased expression (1% xylose compared to 0.1% xylose supplementation) of *hapX* alleles with combinatorial mutation of at least CRR-B and CRR-C increases the growth inhibitory effect in contrast to the native *hapX* allele. Contrary to the growth analysis on solid media (Fig. 1), the growth analysis in liquid media showed a better growth of strain *hapX^B/C^* compared to strain *hapX^A/B/C/D^*, indicating that mutation of CRR-A and/or CRR-D, in addition to CRR-B and CRR-C, plays a role in growth inhibition. In addition, growth characterization in liquid media indicated that combinatorial mutation of at least CRR-B and CRR-C is not lethal, as growth was observed for each allele analyzed.

### Expression of *hapX* alleles with combinatorial mutation of at least CRR-B and -C increases cellular iron accumulation

**Figure 7.**
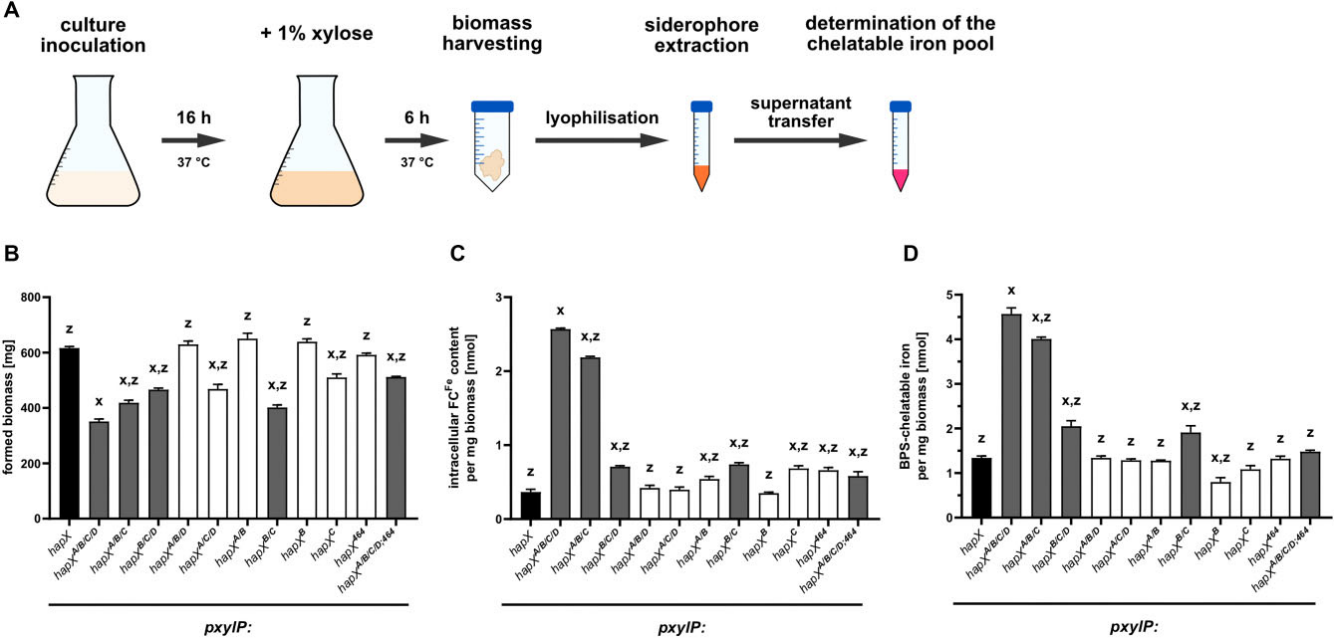
Combinatorial mutation of CRR-B and -C (strains *hapX^A/B/C/D^*, *hapX^A/B/C^*, *hapX^B/C/D^* and *hapX^B/C^*) reduces biomass formation in liquid culture upon xylose supplementation and increases both cellular ferricrocin-complexed iron and the chelatable iron pool. The schematic experimental setup is shown in (**A**). Liquid cultures were inoculated with the respective fungal strains and grown for 16 h at 37 °C in +Fe conditions, followed by supplementation with 1.0% xylose for *pxylP* induction and continued incubation for additional 6 h. After 22 h of incubation, the harvested biomass was used for estimation of biomass formation (**B**) and subsequently for the extraction of ferricrocin-complexed iron (**C**) and the determination of the chelatable iron pool (**D**). Values shown represent the mean ± standard deviation of biological triplicates. Significant differences, calculated by a two-way analysis of variance (ANOVA), are indicated by “*x*” for the reference strain *hapX* and “*z*” for the reference strain *pxylP:hapX^A/B/C/D^* with *P* < 0.0001.

The transcriptomic analysis indicated that combinatorial mutation of the four CRRs (strain *hapX^A/B/C/D^*) causes upregulation of genes involved in iron uptake (Figs 2–4). To investigate intracellular iron accumulation, the mutant strains expressing the different *hapX* alleles were grown in +Fe liquid culture for 16 h without xylose induction, followed by another 6 h in the presence of xylose to induce expression of the different *pxylP*-driven *hapX* alleles. The schematic experimental setup is shown in Fig. 7A. Optical inspection confirmed comparable biomass formation after 16 h of growth without xylose for all strains, consistent with the growth behavior on solid media (Fig. 1). However, after the additional 6 h xylose induction period, all strains with combinatorial mutation of CRR-B and -C (*hapX^A/B/C/D^*, *hapX^A/B/C^*, *hapX^B/C/D^* and *hapX^B/C^*; *hapX^A/B/C/D;464^*), as well as *hapX^A/C/D^* and *hapX^C^* strains, showed significantly reduced biomass formation compared to *hapX^A/B/D^*, *hapX^A/B^*, *hapX^B^*, *hapX^464^* and *hapX* (Fig. 7B). These results further highlight the importance of HapX CRR-B and CRR-C for the fitness of *A. fumigatus* as seen in the plate and liquid growth assays (Figs 1 and 6). Among the strains with combinatorial mutation of CRR-B and -C, strain *hapX^A/B/C/D^* showed the most severe growth defect followed by *hapX*^*B/C*^,*hapX^A/B/C^* and *hapX^B/C/D^*. The statistically significant differences between the biomasses of these strains (Fig. 7B) indicate a function of CRR-A and CRR-D, in addition to CRR-B and CRR-C, which was not seen in the plate growth analyses (Fig. 1) but indicated by the liquid growth assays (Fig. 6). The *hapX^A/B/C/D;464^* strain displayed significantly increased biomass formation compared to the *hapX^A/B/C/D^* strain (Fig. 7B), which is in agreement with the rescue of the growth inhibitory effect seen in the plate growth assays (Fig. 1).

Further analysis revealed that combinatorial mutation of CRR-B and -C increases cellular levels of iron complexed by the intracellular siderophore ferricrocin, a previously shown indicator of cellular iron accumulation [15, 76, 77]: ∼7-fold in *hapX^A/B/C/D^*, ∼6-fold in *hapX^A/B/C^* and ∼2-fold in *hapX^B/C/D^* and *hapX^B/C^* compared to the *hapX* strain (Fig. 7C). The statistically significant differences between these strains again indicate a function of particularly CRR-A but also CRR-D, in addition to CRR-B and CRR-C. C-terminal truncation of HapX (strain *hapX^A/B/C/D;464^*) significantly reduced the accumulation of ferricrocin-complexed iron caused by combinatorial mutation of the four CRRs (strain *hapX^A/B/C/D^*). The individual mutation of CRR-C (*hapX^C^*), combinatorial mutation of CRR-A and -B (*hapX^A/B^*), and C-terminal truncation (*hapX^464^*) also resulted in slightly increased intracellular accumulation of ferricrocin-complexed iron (Fig. 7C).

Another indicator of cellular iron overload is the chelatable iron pool, i.e. cellular iron not complexed by siderophores, which can be measured photometrically by reddish coloration due to chelation by a chelator such as BPS after reduction by ascorbic acid to ferrous iron [24]. Only strains expressing *hapX* alleles with combinatorial mutation of CRR-B and -C showed significantly increased chelatable iron pools with *hapX^A/B/C/D^* > *hapX*^*A/B/C*^> *hapX^B/C/D^* = *hapX^B/C^* (Fig. 7D). Again, the statistically significant differences between these strains indicate a function of particularly CRR-A but also CRR-D, in addition to CRR-B and CRR-C. Again, the C-terminal truncation (strain *hapX^A/B/C/D;464^*) significantly decreased the chelatable iron pool caused by combinatorial mutation of the four CRRs (strain *hapX^A/B/C/D^*). Remarkably, individual mutation of CRR-B reduced the chelatable cellular iron pool (Fig. 7D).

Taken together, the increased levels in ferricrocin-complexed iron and chelatable iron in strains expressing *hapX* alleles with combinatorial mutation of CRR-B and -C are consistent with increased high-affinity iron uptake, as suggested by the gene expression analyses (Figs 2–4), with additional mutation of CRR-A and/or CRR-D amplifying the effect caused by combinatorial mutation of CRR-B and CRR-C alone (Fig. 7).

To distinguish whether combinatorial mutation of the four CRRs blocks growth or is lethal, strains *hapX* and *hapX^A/B/C/D^* were grown in +Fe liquid cultures without xylose supplementation for 16 h to circumvent the growth inhibitory effect of combinatorial mutation of all four CRRs, followed by 12 h growth in the presence of 1% xylose to induce the *pxylP*-driven *hapX* alleles, and biomass formation was determined. Alternatively, the two strains were grown under the same condition, followed by washing the mycelia to remove the xylose and incubated without xylose supplementation for another 12 h (Supplementary Fig. S5). The fact that the biomass formation of strain *hapX^A/B/C/D^* increased significantly during growth without xylose supplementation after washing the mycelia indicates that the combinatorial mutation of the four CRRs inhibits growth but is not lethal, which was also indicated by the liquid growth assay (Fig. 6).

### The 27 amino acid C-terminal region of HapX contains a degron

**Figure 8.**
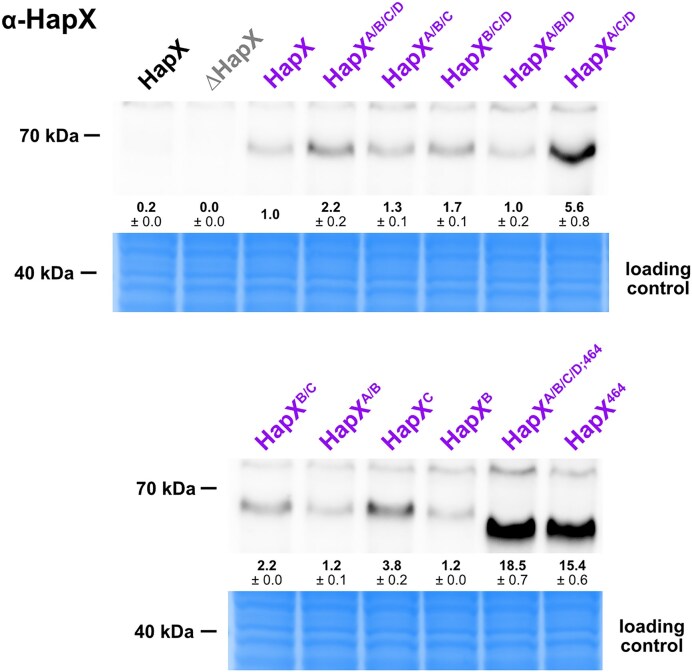
Several mutations of CRRs, and in particular C-terminal truncation, increase the HapX protein level. Strains were grown in +Fe without xylose for 17 h followed by 1 h incubation with 0.1% xylose for induction of *hapX* expression (same mycelia as used for northern blot analysis shown in Fig. 4) and subjected to western blot analysis using an α-HapX antibody. All strains with *pxylP*-driven *hapX* alleles are shown in purple; HapX lacking *ΔhapX* in grey serves as the negative control proving antibody specificity. Production of HapX from the native promoter (WT strain) is shown in black. Densitometrically estimated protein amounts were normalized to *pxylP*-driven WT-like HapX; numbers represent the mean ± standard deviation. Coomassie-stained SDS–polyacrylamide gels are shown as loading controls.

**Figure 9.**
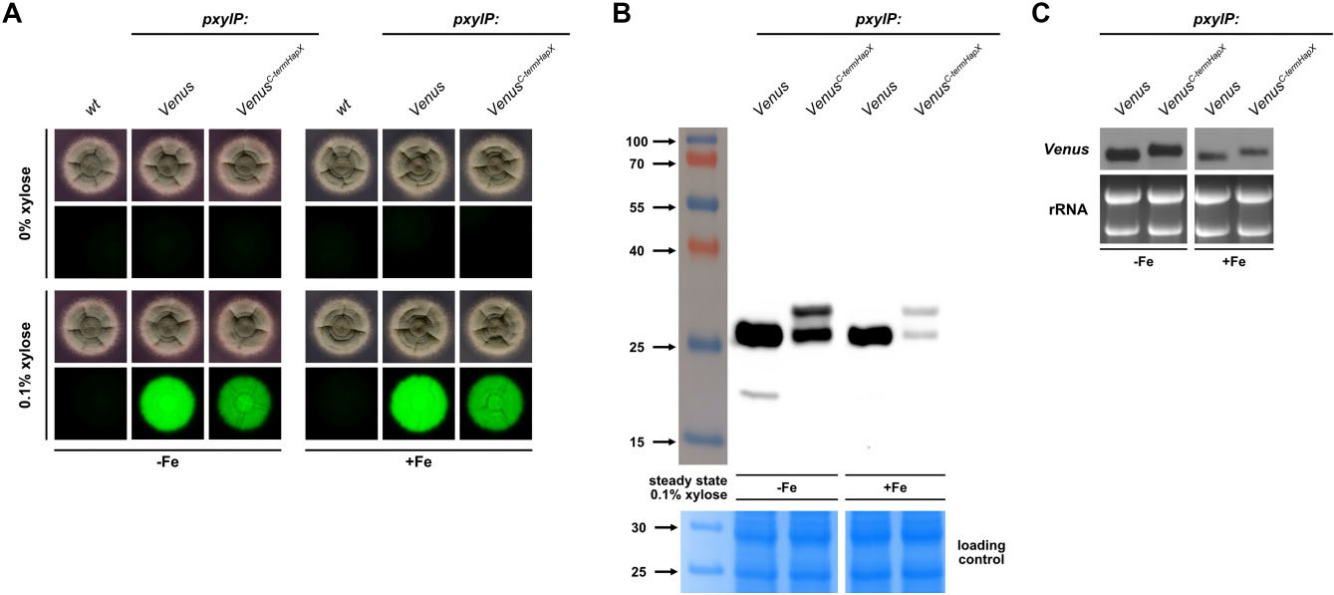
The HapX C-terminus contains a degron. C-terminal fusion of Venus with the C-terminal 27 amino acid region of HapX (strain *Venus^C-termHapX^*) decreases Venus-derived fluorescence intensity of fungal colonies (**A**) and protein stability in western blot analysis (**B**), but does not affect transcript stability (**C**). Solid (**A**) and liquid (**B**) media contained 0.1% xylose for induction of *pxylP*-driven *Venus* alleles. On solid media, 10^4^ conidia were spot-inoculated on medium reflecting iron limitation (−Fe; 200 μM BPS) or iron sufficiency (+Fe; 30 μM FeSO_4_) and incubated at 37 °C for 48 h (**A**). For western blot analysis (**B**) using an α-GFP antibody (Venus *M_r_* = 26.8 kDa; Venus^C-termHapX^*M_r_* = 30.3 kDa), fungal strains were grown for 17 h in −Fe and +Fe liquid culture; −Fe did not contain BPS. Coomassie-stained SDS–polyacrylamide gels are shown as loading controls. Northern blot analysis was performed with a Venus-specific probe. Ethidium bromide-stained rRNA is shown for loading and quality control.

To analyze the consequences of the described mutations and C-terminal truncation on HapX protein abundance, we performed western blot analysis in +Fe without xylose induction to allow growth of all mutant strains followed by a 1 h incubation in the presence of 0.1% xylose to induce expression of *pxylP*-driven *hapX* alleles (Fig. 8). As reported previously [10, 21], HapX was barely detectable in WT under +Fe conditions and was absent in the *ΔhapX* strain, while *pxylP* control increased the HapX protein level (Fig. 8). Compared to the WT-like HapX (HapX), the different CRR mutations resulted in similar or slightly higher HapX protein levels, with the combinatorial mutation of CRR-A, -C, and -D (HapX^A/C/D^) resulting in the highest increase, ∼6-fold, indicating increased stability of this HapX version as expression of all versions was driven by the same promoter (Fig. 8). Remarkably, the truncation of the C-terminal 27 amino acid region, with (HapX^A/B/C/D;464^) or without (HapX^464^) combinatorial mutation of all four CRRs, increased the HapX protein levels ∼10-fold (Fig. 8), suggesting that the truncated region may contain a degron, a protein domain regulating the protein’s half-life by mediating its degradation [78]. In agreement, fusion of the C-terminal 27 amino acid region of HapX to *pxylP*-driven Venus (strain *Venus^C-termHapX^*) significantly reduced Venus protein levels (Venus *M_r_* = 26.8 kDa; Venus^C-termHapX^*M_r_* = 30.3 kDa) in both −Fe and +Fe, as judged by both Venus-derived fluorescence of fungal colonies (Fig. 9A) as well as the Venus protein levels in western blot analysis (Fig. 9B), but did not affect the transcript level (Fig. 9C). Moreover, western blot analysis clearly demonstrated degradation of the Venus-HapX fusion protein with the major degradation product having a molecular mass similar to that of Venus. Notably, Venus, like other green fluorescent protein relatives, forms an exceptionally stable 11-stranded β-barrel, which is difficult to degrade leading to quite stable intermediates that are still fluorescent [79]. Therefore, the Venus-sized protein may be a more stable degradation intermediate.

The functions of the 27 amino acid C-terminal region of HapX in protein stability and iron starvation adaptation appear to be independent as overexpression of the *hapX^A/B/C/D^* allele did not rescue the growth defect on solid media (Supplementary Fig. S6) and exacerbated the growth defect in liquid media (Fig. 6).

### The 27 amino acid C-terminal region of HapX contains an evolutionarily conserved sequence

Multiple alignments of HapX homologs from different Ascomycota lineages (Eurotiomycetes, Lecanoromycetes, Leotiomycetes, Pezizomycetes, Sordariomycetes, and Pichiomycetes) showed conservation not only of the N-terminal DNA-binding and CCAAT complex-interacting regions, the four CRRs, but also of two C-terminal regions, here termed conserved C-terminal regions 1 and 2, CCTR1 and CCTR2 (Supplementary Fig. S7). CCTR1, localized in the 27 amino acid C-terminal region, comprises about 18 amino acid residues (in *A. fumigatus*: ^471^EVEAASVVMGVLRYFDRRF^488^), of which 17 are conserved in most Eurotiomycetes and 10 in HapX homologs from different Ascomycota lineages (Supplementary Fig. S7). CCTR2, which precedes CCTR1 N-terminally, consists of approximately 30 amino acid residues (in *A. fumigatus*: ^419^TLSCADAYTTLSRHPNFSRATDELSTWLPK^448^), of which 15 are conserved in most homologs from different Ascomycota lineages (Supplementary Fig. S7). Truncation of the C-terminal 27 amino acid residues (HapX^464^ mutant strains) eliminates CCTR1 but not CCTR2.

The crystal structure of the N-terminal part of the *A. nidulans* HapX homolog containing the DNA-binding and CCAAT binding complex interacting domains has been reported [22], but the structure of the downstream region containing the CRRs remains unresolved. The AlphaFold2 prediction (AF-Q4WER3-F1) [51, 52] of *A. fumigatus* HapX (Supplementary Fig. S8A) suggests that CCTR1 folds into an α-helix that interacts with the largely α-helical CCTR2, which is in close contact with an α-helix overlapping CRR-C. Analysis by ConSurf web server (https://consurf.tau.ac.il) [80] suggested that the amino acid residues that make up this structure are evolutionarily highly conserved in HapX homologs (Supplementary Fig. S8B). Apart from CCTR1, CCTR2, and short α-helices N-terminally overlapping CRR-A, -B, and -C, the HapX region downstream of the DNA-binding domain largely lacks structural prediction. In addition, [2Fe-2S] cluster binding by the CRRs is expected to cause structural changes of the structure.

Taken together, these data suggest that the functions mediated by the 27 amino acid C-terminal region, adaptation to iron starvation and/or protein stability, are evolutionarily conserved.

## Discussion

Previous studies suggested that HapX senses the cellular iron state by sensing the abundance of cellular [2Fe-2S] clusters [4, 10, 17, 23, 24, 81]. Consistent with this, mutation of mainly CRR-B, but to a lesser extent also CRR-A and even less CRR-C impaired hFe adaptation of *A. fumigatus*, while mutation of CRR-D had no effect [14]. These results underline a role for three CRRs in the sensing of hFe by HapX. However, how HapX senses −Fe remained elusive, as individual mutation of any of the four CRRs did not affect −Fe adaptation of *A. fumigatus* [14].

Here, systematic combinatorial mutation of the HapX CRRs using conditional expression of the respective alleles with the xylose-inducible *xylP* promoter [34, 35] revealed that expression of *hapX* alleles with combinatorial mutation of at least CRR-B and CRR-C (*hapX^A/B/C/D^*, *hapX^A/B/C^*, *hapX^B/C/D^* and *hapX^B/C^*), but not of other combinatorial CRR mutations (*hapX^A/B/D^*, *hapX^A/C/D^* and *hapX^A/B^*), nor individual mutation of CRR-B (*hapX^B^*) or CRR-C (*hapX^C^*), blocks growth on solid media regardless of iron availability, i.e. in −Fe, +Fe, and hFe conditions (Fig. 1). Transcriptome and individual gene expression analyses (Figs 2–4) revealed that combinatorial mutation of CRR-B and CRR-C results in a transcriptional −Fe response in +Fe, i.e. the induction of genes involved in siderophore-mediated iron acquisition [15, 17] and the repression of all 33 previously identified HapX target genes involved in iron-consuming pathways [12]. Taken together, these results suggest that combinatorial mutation of CRR-B and CRR-C locks HapX in the −Fe state. In other words, at least CRR-B and CRR-C, and possibly also CRR-A and -D, lack [2Fe-2S] in −Fe. Consequently, [2Fe-2S] cluster occupancy of CRR-B and/or -C switches HapX out of the −Fe state. Remarkably, of all four CRRs, CRR-B coordinates a [2Fe-2S] cluster with particularly high stability, by far higher than the other three CRRs [23]. Therefore, CRR-B may be the first CRR sensing iron availability by binding a [2Fe-2S] cluster followed by CRR-C. As suggested by the growth defects in hFe caused by individual mutation of CRR-A, -B, and -C with endogenous promoter control [14] and with conditional expression (Fig. 1), the switching of HapX to the hFe state then requires the binding of a [2Fe-2S] cluster also by CRR-A. Notably, in all plate growth assays, mutation of CRR-D was phenotypically inconspicuous in all combinations tested.

In plate assays, all strains expressing *hapX* alleles with combinatorial mutations of at least CRR-B and -C blocked growth in all combinations (Fig. 1). In contrast, in liquid growth assays, combinatorial mutation of all four CRRs had a more severe growth inhibitory effect compared to combinatorial mutation of only CRR-B and -C (Fig. 6). In line, induction of different *hapX* alleles after uninduced pre-growth in liquid media revealed differences (Fig. 7B): Strain *hapX^A/B/C/D^* showed the most severe growth defect followed by *hapX*^*B/C*^,*hapX^A/B/C^* and *hapX^B/C/D^*. Further analysis of these strains revealed that combinatorial mutation of CRR-B and -C increases cellular iron accumulation, i.e. it increased the cellular levels of iron complexed by the intracellular siderophore ferricrocin with *hapX^A/B/C/D^* > *hapX*^*A/B/C*^> *hapX^B/C/D^* > *hapX^B/C^* (Fig. 7C) as well as the chelatable iron pool in a similar order with *hapX^A/B/C/D^* > *hapX*^*A/B/C*^> *hapX^B/C/D^* ≈ *hapX^B/C^* (Fig. 7D). The statistically significant differences in biomass formation, ferricrocin-complexed iron, and the chelatable iron pool between strains *hapX^A/B/C/D^*, *hapX*^*B/C/D*^,*hapX^A/B/C^* and *hapX^B/C^* indicate that additional mutation of particularly CRR-A but also CRR-D enhances the effect caused by combinatorial mutation of CRR-B and CRR-C alone, which was not seen in the plate growth assays (Fig. 1). Remarkably, individual mutation of CRR-B and, to a lesser extent, CRR-C reduced the chelatable cellular iron pool (Fig. 7D). Both ferricrocin-complexed iron and the chelatable iron pool are previously shown indicators for iron overload [15, 24, 76, 77]. The increased cellular iron accumulation in strains expressing *hapX* alleles with combinatorial mutation of CRR-B and -C is consistent with the increased expression of genes involved in siderophore-mediated iron acquisition (Figs 2–4). Notably, all strains that showed an increased chelatable iron pool (Fig. 7D) featured combinatorial mutation of at least CRR-B and -C and failed to grow on solid media regardless of iron availability (Fig. 1), i.e. an increased chelatable iron pool was the best indicator of a growth defect in this experimental setup. This may be explained by the fact that the chelatable iron pool has a high potential to cause oxidative stress via Haber–Weiss/Fenton chemistry, whereas chelation of iron by siderophores is thought to prevent the Fenton reaction [7, 82]. Similar to strains expressing *hapX* alleles with combinatorial mutation of CRR-B and -C, depletion of the desulfurase Nfs1, which impairs mitochondrial [2Fe-2S] cluster biosynthesis and consequently iron sensing, was shown to result in a transcriptional −Fe response in combination with an increase in both ferricrocin-complexed iron and chelatable iron [24]. These results underscore that combinatorial mutation of CRR-B and -C impairs iron sensing via [2Fe-2S] cluster perception.

Previously, the C-terminal 27 amino acid residues of HapX (amino acid residues 465–491) have been shown to be crucial for adaptation to −Fe but not to hFe [14], i.e. their truncation impaired growth, siderophore biosynthesis, and repression of iron-consuming pathways in −Fe. Truncation of this C-terminal region (strain *hapX^A/B/C/D;464^*compared to strain *hapX^A/B/C/D^*) partially rescued the growth defect caused by mutation of the four CRRs (Fig. 1), attenuated the transcriptional −Fe response (Fig. 5), and reduced both ferricrocin-complexed iron (Fig. 7C) and the chelatable iron pool (Fig. 7D). These results underline that the growth defect caused by combinatorial mutation of the four CRRs is indeed caused by the locked HapX −Fe state resulting in activation of high-affinity iron uptake combined with repressed iron consumption due to HapX-mediated repression of genes involved in iron-consuming pathways, including repression of CccA-mediated iron detoxification by vacuolar iron deposition [13], leading to cellular iron overload and cellular stress. Consequently, the most likely reason for the growth inhibitory effect of combinatorial mutation of at least CRR-B and -C is based on the fact that iron is both essential for growth and toxic when mismanaged, as is the case of mutants expressing HapX alleles that are locked in the iron starvation state and unable to adapt to the cellular iron status. Consistent with this, the growth defect in liquid media caused by combinatorial mutation of at least CRR-B and -C was more severe in +Fe compared to −Fe (Fig. 6). Constitutive expression of *cccA*, which mediates iron detoxification by vacuolar iron deposition [13, 14] and is repressed by the *hapX^A/B/C/D^* allele (Fig. 3A), slightly rescued the growth defect caused by the *hapX^A/B/C/D^* allele (Supplementary Fig. S3). This indicates that CccA-mediated iron detoxification plays a role in the growth defect, but is not the sole cause. Therefore, the constitutive repression of several iron-consuming pathways that are essential for growth and of genes that are important for detoxification of oxidative and nitrosative stress (Fig. 3C) also plays an important role in the growth defect. Consistently, combinatorial mutation of all CRRs caused transcriptional upregulation of several genes encoding stress-responsive regulatory and structural proteins and indicated repression of TOR complex 1 (TORC; target of rapamycin), a nutrient-sensitive, central regulator of cell growth. Starvation, stress, and the drug rapamycin repress TORC1, resulting in repression of protein synthesis, induction of autophagy, exit from the cell cycle, and entry into a quiescent G0 state [65, 66]. Therefore, repression of TORC1 may also be involved in growth inhibition by combinatorial mutation of at least CRR-B and -C.

Western blot analysis indicated that the C-terminal 27 amino acid residues contain a degron (Fig. 8), which was confirmed by expression of Venus, tagged with the C-terminal 27 amino acid region of HapX, in *A. fumigatus* (Fig. 8A). Previously, the HapX protein was shown to have a very short half-life of about 15 min in −Fe, to be post-translationally modified by ubiquitination, SUMOylation, and phosphorylation, and to physically interact with the monothiol glutaredoxin GrxD and the F-box protein Fbx22 [17, 37]. This newly identified degron represents a new factor influencing the protein stability of HapX and illustrates the complex post-translational regulation of this transcription factor. Comparison of HapX homologs from different Ascomycota lineages revealed two evolutionarily conserved regions: CCTR1, located in the C-terminal 27 amino acids, and CCTR2, located N-terminally adjacent to CCTR1 (Supplementary Fig. S7). These results may indicate the evolutionary conservation of the degron and/or the function in iron starvation adaptation. These two functions appear to be independent as overexpression of *hapX^A/B/C/D^* did not rescue the growth defect on solid media (Supplementary Fig. S6) and even accelerated the growth defect in liquid media (Fig. 6). The importance of C-terminal 27 amino acid region for iron starvation adaptation may be based on its structural requirement, such as intramolecular interaction with CCTR2 and via CCTR2 with CRR-C, as suggested by AlphaFold2 prediction (Supplementary Fig. S8A).

Interestingly, the transcriptome analysis also indicated that combinatorial mutation of all CRRs causes upregulation of several gene clusters for biosynthesis of secondary metabolites, i.e. fumarylalanine, pyomelanin/tyrosine degradation, helvolic acid, fumigermin, meroterpenoid pyripyropene, fumitremorgins, and fumagillin/pseurotin (Fig. 3E). These gene clusters may be directly activated by the −Fe state of HapX, by the general −Fe response, or by the induced stress that ultimately blocks growth. Notably, the induction of fumarylalanine and pyomelanin/tyrosine degradation has previously been linked to cellular stress [68, 69].

**Figure 10.**
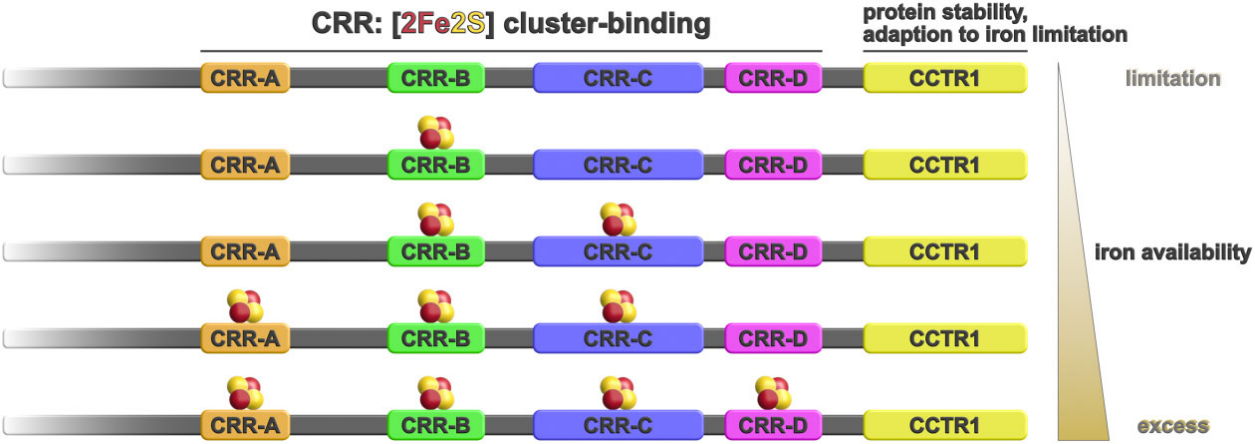
Proposed model for iron sensing by the *A. fumigatus* regulator HapX. The figure illustrates the HapX region containing the phylogenetically conserved CRRs and CCTR1. Previous studies have suggested that HapX switches between three states in order to adapt to varying iron availability. According to the proposed model, the HapX state transitions are regulated by the different propensities of the four CRRs to coordinate [2Fe-2S] clusters, resulting in cumulative occupancy depending on iron availability. The iron starvation state features no [2Fe-2S] clusters in any of the CRRs; the iron sufficiency/“neutral” state features clusters in CRR-B and/or CRR-C; and the iron excess state features clusters in CRR-A, CRR-B, and CRR-C, while CRR-D plays a minor role. Combinatorial mutation of CRR-B and CRR-C locks HapX in the iron starvation state, indicating that these domains are the first to sense [2Fe-2S] clusters. The extraordinarily high stability of [2Fe-2S] cluster coordination by CRR-B suggests that this domain is likely to be the first to bind a [2Fe-2S] cluster in response to iron sensing. The sequential coordination of [2Fe-2S] clusters by the CRRs is expected to result in structural changes that mediate the different HapX functions during iron starvation, sufficiency, and excess. CCTR1 contains a degron and plays an important role in the function of HapX during iron starvation, but not during iron excess, presumably for structural reasons and potentially through interaction with CCR-C via the CCTR2 domain, as suggested by AlphaFold2 prediction of the HapX protein structure. Loss of HapX blocks growth under iron excess and decreases growth under iron starvation but has no visible effect under iron sufficiency. In contrast, combinatorial mutation of CRR-B and CRR-C causes a drastic growth defect under all three conditions. Consequently, the constitutive activation of iron acquisition combined with constitutive repression of iron-consuming pathways and iron detoxification, caused by combinatorial mutation of CRR-B and CRR-C is more detrimental than the de-repression of iron-consuming pathways and reduction of iron acquisition during iron starvation caused by loss of HapX.

Taken together, the present study suggests a model for the switching of HapX between its three functional states by the different propensities of the four CRRs for [2Fe-2S] cluster coordination dependent on iron availability (Fig. 10). In a living cell, there are multiple HapX molecules that are unlikely to all be in the same functional state, allowing fine-tuning of regulation through the distribution of the three functional states. As previously reported [14, 23] and illustrated in Supplementary Fig. S7, most of the HapX homologs from filamentous ascomycetes comprise several CRRs, with CRR-B being the most highly conserved one, suggesting a similar mechanism to *A. fumigatus* HapX. However, there are transcription factors that share only some domains and thus not all functions with HapX: *S. cerevisiae* Yap5 has only two [2Fe-2S] cluster-coordinating CRRs, one of which is highly similar to HapX-CRR-B (67% identity at the amino acid level) and is required exclusively for hFe resistance [83]. In contrast, *Schizosaccharomyces pombe* Php4, which is involved exclusively in adaptation to −Fe, lacks any classical CRR and contains two cysteine residues for binding of a bridging [2Fe-2S] cluster in cooperation with a monothiol glutaredoxin [4, 84]. This data illustrates the modular toolbox used in iron-regulatory transcription factors and underlines the requirement of multiple CRRs for transcription factors enabling adaptation to both −Fe and hFe conditions.

## Supplementary Material

gkaf796_Supplemental_Files

## Data Availability

Raw data generated in this study have been deposited at GEO under the accession number GSE286351.
