# Supplementary material for: Functional transitions of the Aspergillus fumigatus iron regulator HapX are governed by conserved domains cooperatively binding [2Fe-2S] clusters
Source: Nucleic Acids Res. 2025 Aug 27;53(16):gkaf796. doi: 10.1093/nar/gkaf796 (PMC12390756; doi:10.1093/nar/gkaf796)
Supplement: gkaf796_Supplemental_Files [file gkaf796_supplemental_files.zip › 2025_ObereggerS_HapX_Supplementary_Material.pdf]

SUPPLEMENTARY INFORMATION

Functional transitions of the *Aspergillus fumigatus* iron regulator HapX are governed by conserved domains cooperatively binding [2Fe-2S] clusters

Simon Oberegger[1], Matthias Misslinger[1], Isidor Happacher[1] and Hubertus Haas[1*]

[1] Institute of Molecular Biology, Biocenter, Medical University Innsbruck, Innsbruck, Austria

[*] corresponding author: hubertus.haas@i-med.ac.at

ABBREVIATIONS

BPS, bathophenanthroline disulfonic acid; **CCTR**, conserved C-terminal regions; **CRR**, cysteine-rich region; **-Fe**, iron-limiting condition; **+Fe**, iron sufficient condition; **hFe**, iron excess condition; ***pxylP***, xylose-inducible *xylP* promoter; ***wt***, wild type.

SUPPLEMENTARY FIGURES

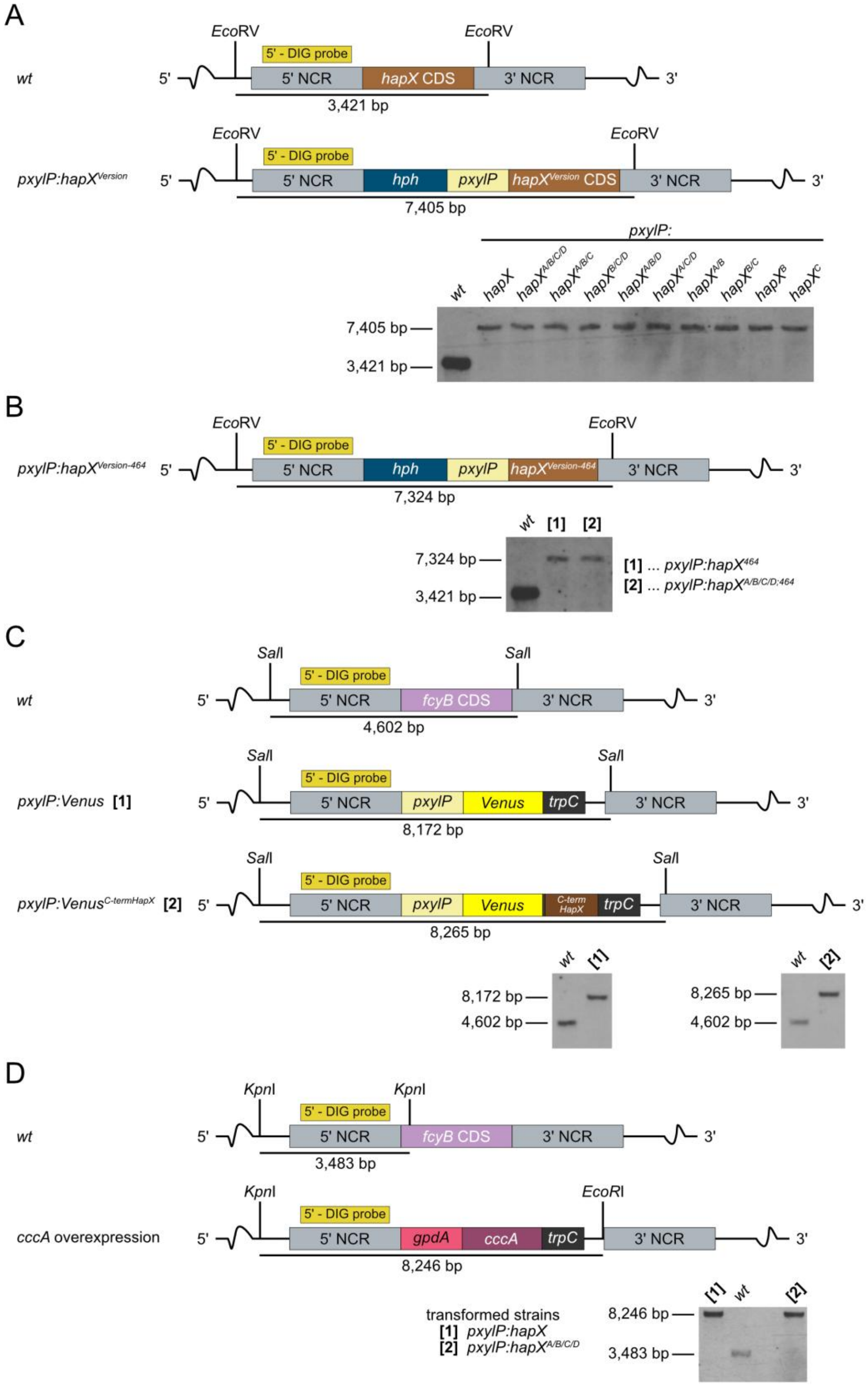

Supplementary Figure S1: Schemes of the targeted genomic loci and Southern blot confirmation of the generated mutant strains. Genomic DNA was digested using specific restriction enzymes and then subjected to Southern blot analysis. **(A)** *hapX* locus in *wt* and transformed *hapX* CRR mutant strains; digestion using *Eco*RV led to a detected fragment length of 3,421 bp in *wt* and in a 7,405 bp fragment in the mutant strains; **(B)** in strains encoding C-terminally truncated *hapX* alleles the detected fragment in the mutant strains was slightly smaller, i.e. 7,324 bp. **(C)** *wt fcyB* locus, used for marker-free integration of the conditionally expressed *Venus* constructs into the *wt* background. *Sal*I digestion resulted in a detected *wt* fragment length of 4,602 bp which increased to 8,172 bp in the *Venus* and to 8,265 bp in the *Venus*$^{C\text{-}termHapX}$ mutant strains, respectively. **(D)** *wt*

fcyB locus, used to overexpress *cccA* under the control of the constitutive promoter *gpdA* in the generated *hapX* mutant strains. *Kpn*I and *EcoR*I digestion resulted in the detection of a *wt* fragment length of 3,483 bp, which increased to 8,246 bp in $cccA^{OE}$ mutant strains. All Southern blot results are in line with the *in silico* predictions.

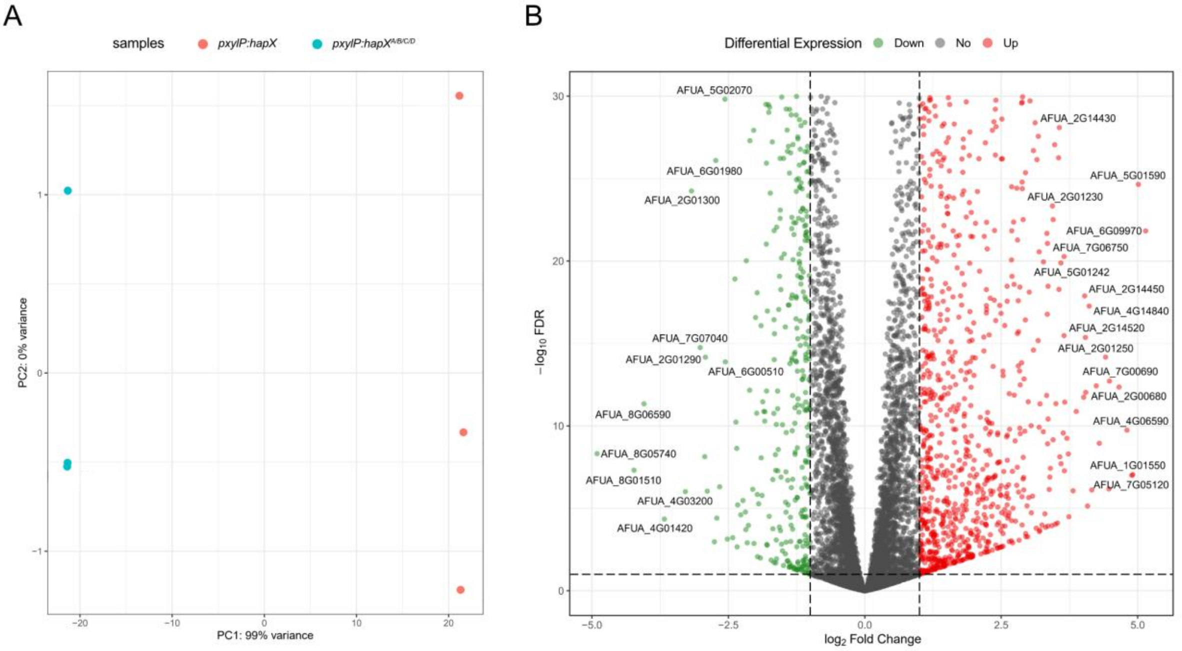

Supplementary Figure S2: Global comparison of gene expression in strains *hapX* and $hapX^{A/B/C/D}$. (A) Principal component analysis showing clustering of biological triplicates and differences between the two strains analyzed. **(B)** Volcano blot analysis illustrating the differences in gene expression in $hapX^{A/B/C/D}$ compared to *hapX*. Genes downregulated with a log2 fold change ≤ -1 and upregulated with ≥ 1 in $hapX^{A/B/C/D}$ compared to *hapX* are highlighted in green and red, respectively. The log2 fold change values are plotted on the x-axis, while the y-axis represents the -log10 false discovery rate (FDR).

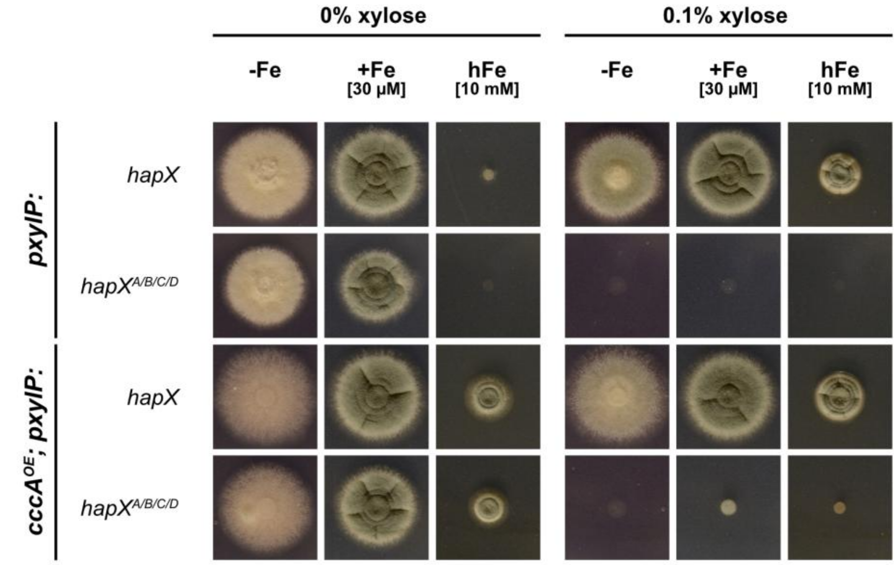

Supplementary Figure S3: Constitutive expression of *cccA* (strain $cccA^{OE}$; $pxylP$:$hapX^{A/B/C/D}$) slightly rescues the growth defect caused by combinatorial mutation of the four CRRs (strain $hapX^{A/B/C/D}$) on +Fe and hFe. For each fungal strain, 10^4 conidia were spot-inoculated on minimal medium plates reflecting iron starvation (-Fe; 200 µM BPS), iron sufficiency (+Fe; 30 µM $FeSO_4$) or iron excess (hFe; 10 mM $FeSO_4$). Expression of *pxylP*-driven *hapX* alleles was tuned by supplementation with xylose: 0% for repression and 0.1% for moderate expression. Plates were incubated at 37 °C for 48 h.

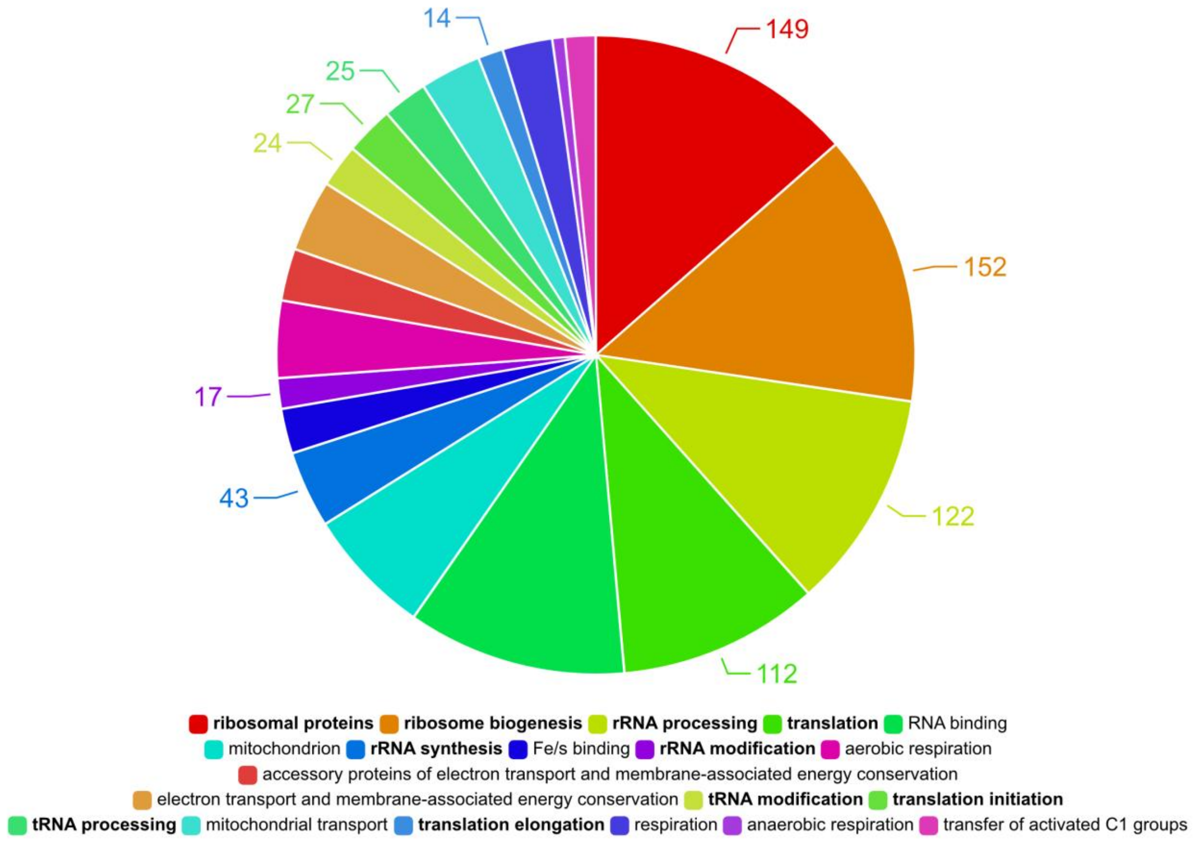

Supplementary Figure S4: The majority of downregulated genes in strain *hapX*$^{A/B/C/D}$ are related to ribosome biogenesis and translation. Functional annotation of significantly downregulated genes in *hapX*$^{A/B/C/D}$ compared to *hapX* was done using FungiFun2 (1). Functional categories linked to ribosome biogenesis and translation are in bold. Respective genes are listed in Supplementary Table S1C.

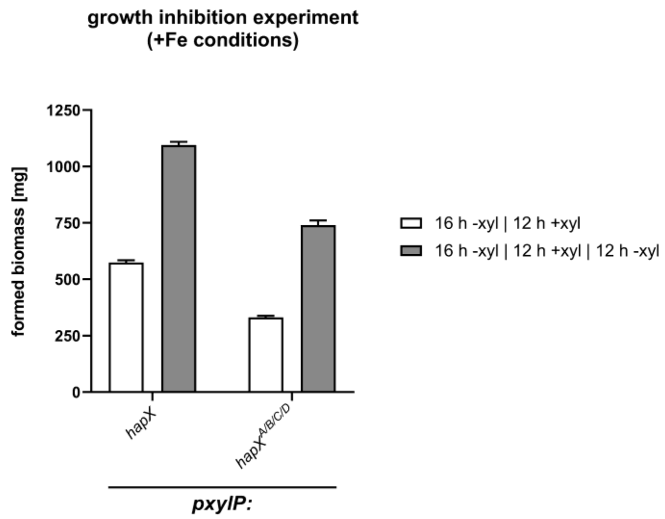

Supplementary Figure S5: Combinatorial mutation of the four CRRs (strains *hapX*$^{A/B/C/D}$) is not lethal. Strains *hapX* and *hapX*$^{A/B/C/D}$ were grown at 37 °C in +Fe liquid cultures without xylose supplementation for 16 h to circumvent the growth inhibitory effect of combinatorial mutation of all four CRRs, followed by 12 h growth in the presence of 1% xylose to induce expression of the *pxylP*-driven *hapX* alleles (16 h -xyl / 12 h + xyl). Alternatively, the two strains were grown under the same conditions, followed by washing of the mycelia to remove the xylose and incubation without xylose supplementation for a further 12 h (16 h -xyl / 12 h + xyl / 12 h -xyl). The biomass formation values shown represent the mean ± standard deviation of biological triplicates. The fact that biomass formation of strain *hapX*$^{A/B/C/D}$ increased significantly during growth without xylose supplementation after washing the mycelia indicates that the combinatorial mutation of the four CRRs inhibits is not lethal.

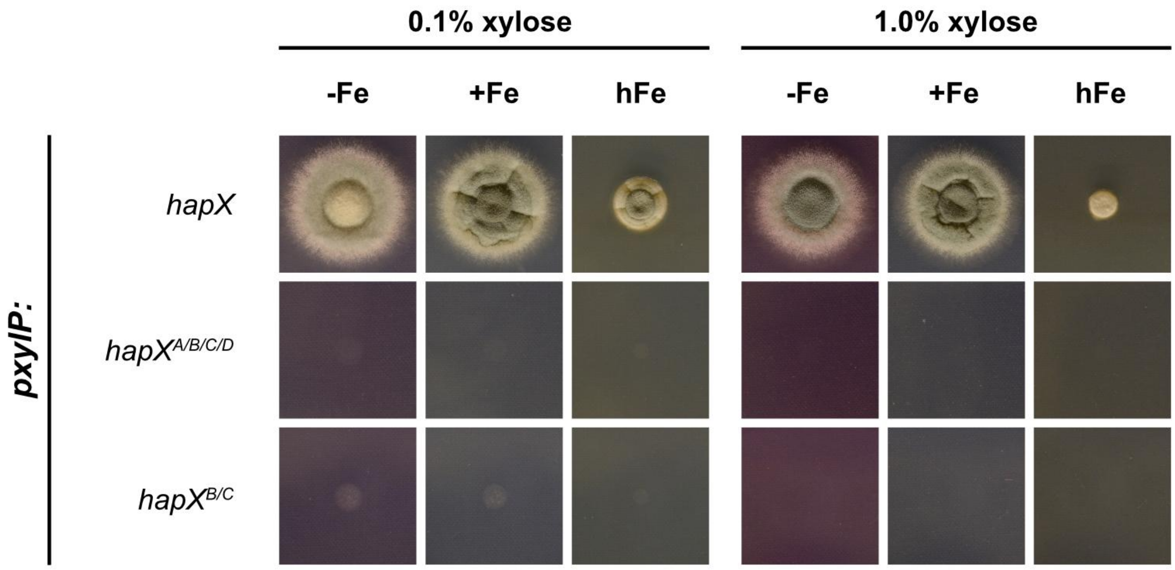

Supplementary Figure S6: Overexpression of *hapX$^{A/B/C/D}$* does not rescue the growth defect caused by combinatorial mutation of the CRRs (strains *hapX$^{A/B/C/D}$*, strains *hapX$^{B/C}$*). For each fungal strain, 10^4 conidia were spot-inoculated on minimal medium plates reflecting iron starvation (-Fe; 200 µM BPS), iron sufficiency (+Fe; 30 µM $FeSO_4$) or excess iron (hFe; 10 mM $FeSO_4$). Expression of *pxylP*-driven *hapX* alleles was induced by supplementation with xylose: 0.1% for moderate expression and 1.0% for overexpression. Plates were incubated at 37 °C for 48 h. Notably, the 0.1% xylose images were included from Figure 1 for comparison.

A

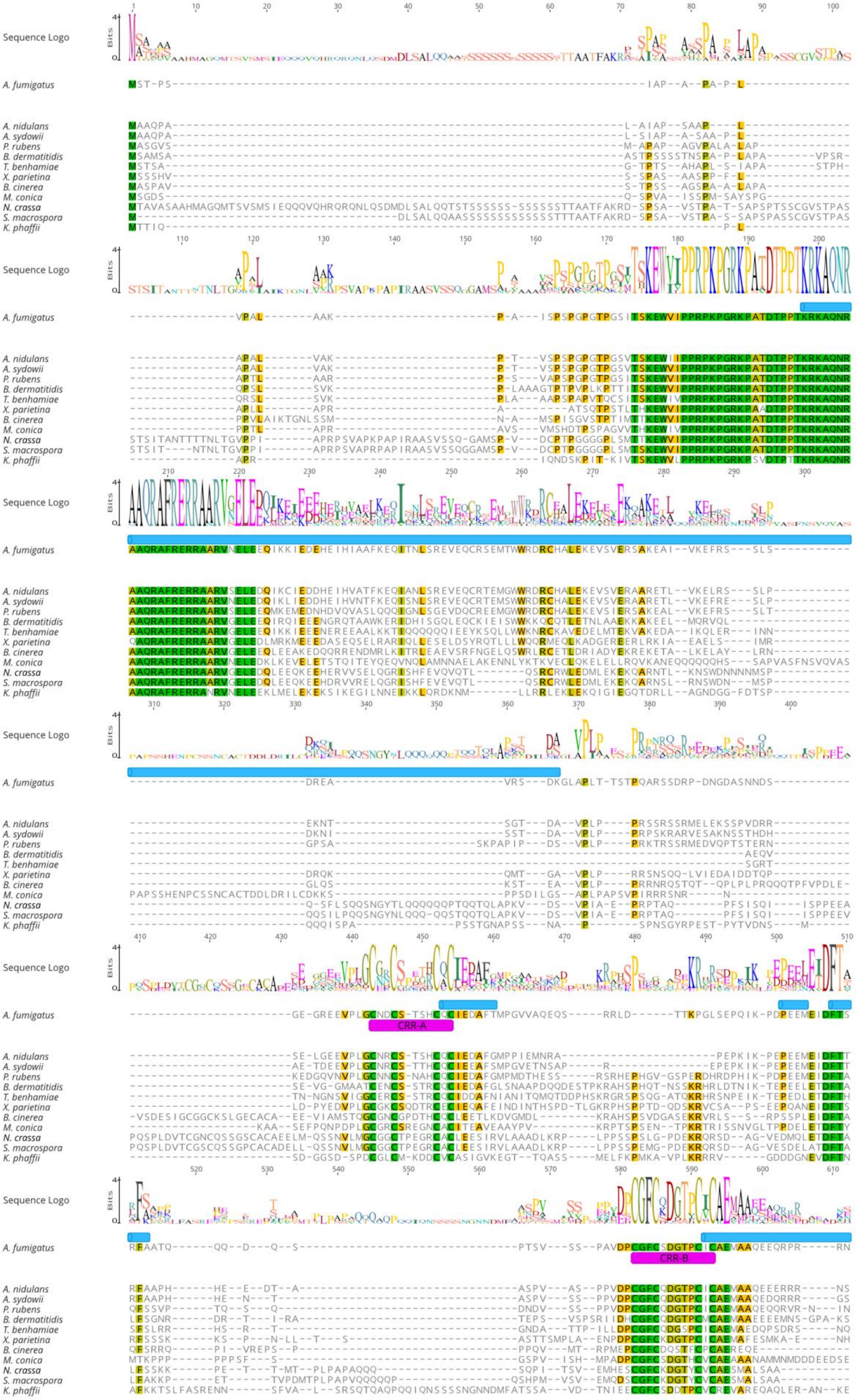
Sequence Logo
Bits
A. fumigatus
A. nidulans
A. sydowii
P. rubens
B. dermatitidis
T. benhamiae
X. parietina
B. cinerea
M. conica
N. crassa
S. macrospora
K. phaffii
CRR-A
CRR-B

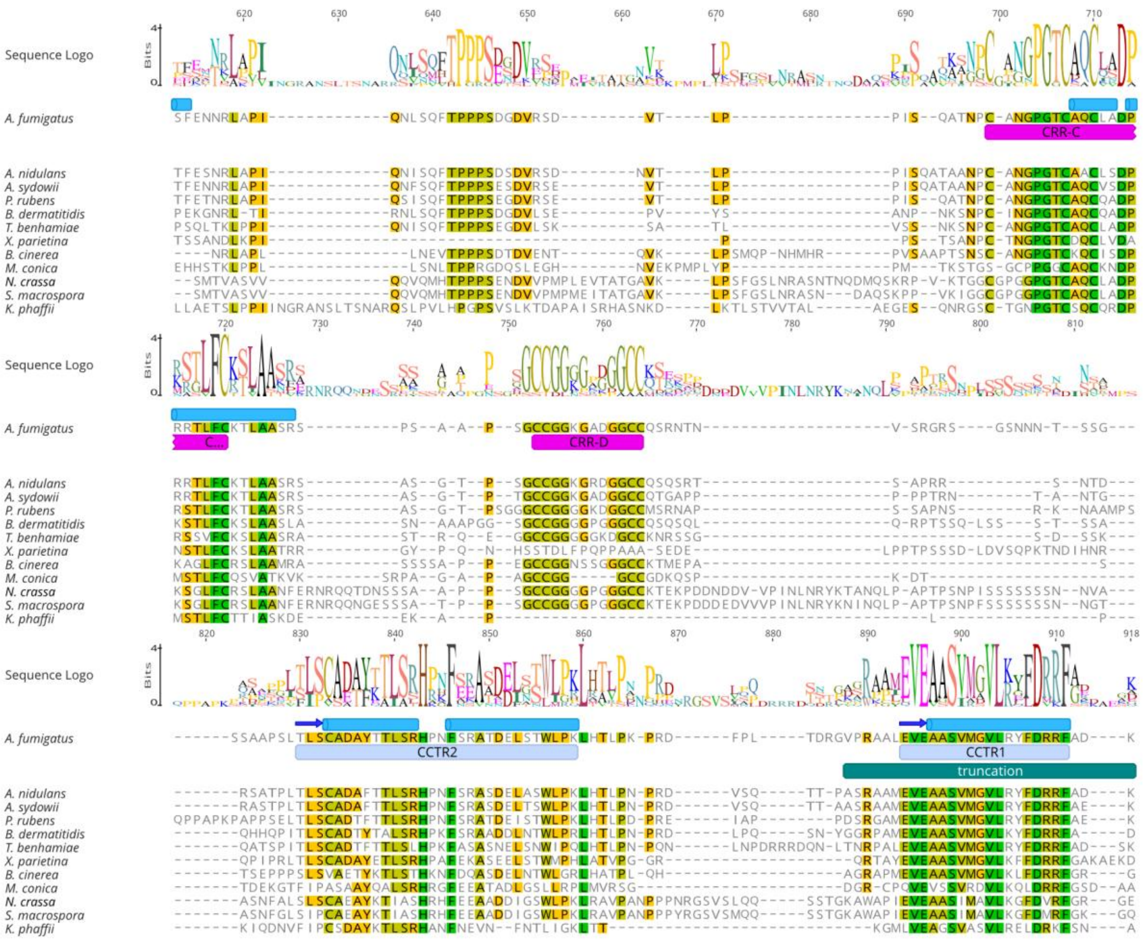

B

Subdivision	Class	Order	Family	Genus	Species	Accession #	E-Value
Pezizomycotina	Eurotiomycetes	Eurotiales	Aspergillaceae	*Aspergillus*	*A. fumigatus*	Afu5g03920	query
Pezizomycotina	Eurotiomycetes	Eurotiales	Aspergillaceae	*Aspergillus*	*Aspergillus nidulans*	XP_681520.1	0
Pezizomycotina	Eurotiomycetes	Eurotiales	Aspergillaceae	*Aspergillus*	*Aspergillus sydowii*	XP_040697762.1	0
Pezizomycotina	Eurotiomycetes	Eurotiales	Aspergillaceae	*Penicillium*	*Penicillium rubens*	XP_002565316.1	0
Pezizomycotina	Eurotiomycetes	Onygenales	Ajellomycetaceae	*Blastomyces*	*Blastomyces dermatitidis*	XP_045276186.1	5.94E-94
Pezizomycotina	Eurotiomycetes	Onygenales	Arthrodermataceae	*Trichophyton*	*Trichophyton benhamiae*	XP_003015051.1	1.33E-100
Pezizomycotina	Lecanoromycetes	Teloschistales	Teloschistaceae	*Xanthoria*	*Xanthoria parietina*	CAL8583898.1	2.68E-72
Pezizomycotina	Leotiomycetes	Helotiales	Sclerotiniaceae	*Botrytis*	*Botrytis cinerea*	XP_001546978.2	4.45E-62
Pezizomycotina	Pezizomycetes	Pezizales	Morchellaceae	*Morchella*	*Morchella conica*	RPB12462.1	4.96E-33
Pezizomycotina	Sordariomycetes	Sordariales	Sordariaceae	*Neurospora*	*Neurospora crassa*	XP_958829.3	2.16E-26
Pezizomycotina	Sordariomycetes	Sordariales	Sordariaceae	*Sordaria*	*Sordaria macrospora*	XP_003346867.2	3.22E-29
Saccharomycotina	Pichiomycetes	Pichiales	Pichiaceae	*Komagataella*	*Komagataella phaffii*	XP_002489509.1	1.5E-31

Supplementary Figure S7: Amino acid alignment of HapX and homologs from different fungal lineages. The protein sequence of *A. fumigatus* HapX (Afu5g03920) was blasted against selected species on the NCBI database (https://blast.ncbi.nlm.nih.gov/Blast.cgi) (2). **(A)** A MUSCLE alignment of the protein sequences was performed using Geneious Prime 2025.0.3 (https://www.geneious.com). The structural predictions of beta sheets (dark blue arrows) and alpha helices (sky blue cylinders), indicated above the *A. fumigatus* sequence, were obtained from the AlphaFold structure (AF-Q4WER3-F1-v4) (3,4). The *A. fumigatus* HapX CRR regions, CRR-A (200-210), CRR-B (277-288), CRR-C (343-363) and CRR-D (379-390), are highlighted in magenta. The two conserved C-terminal regions (CCTR), CCTR1 (471-488) and CCTR2 (419-448), are highlighted in grey-blue and the truncation of HapX is highlighted in turquoise. The similarities of amino acids are indicated using the score matrix Blosum62: green corresponds to 100%, gold corresponds to 80-99% and yellow corresponds to 60-79% amino acid similarity. **(B)** The lineages of the compared species are shown, together with the accession numbers and E-values.

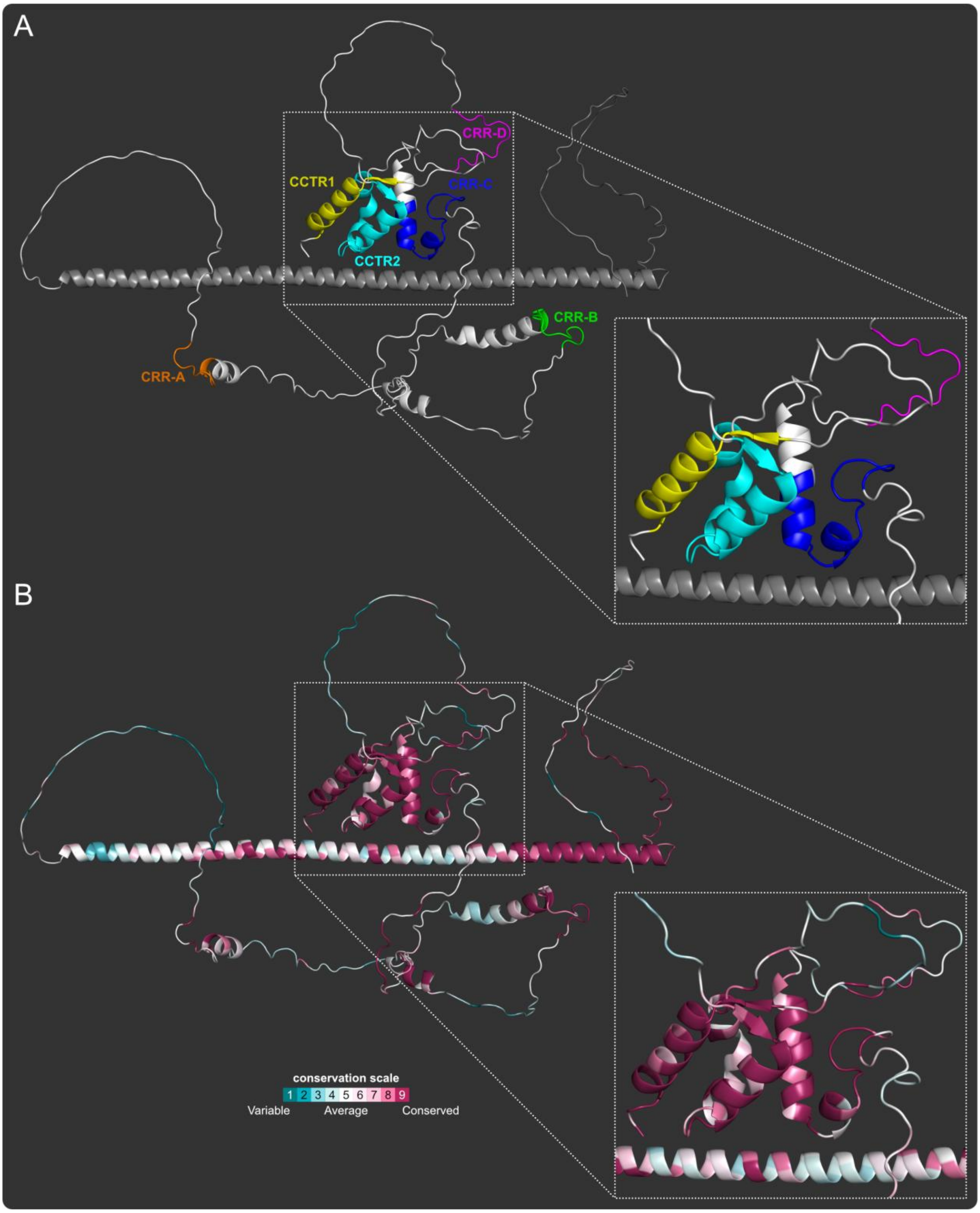

Supplementary Figure S8: Analysis of the structure (A) and evolutionary conservation (B) of HapX regions. (A) The *A. fumigatus* HapX protein structure according to AlphaFold prediction (AF-Q4WER3-F1-v4 (3,4)) is shown in cartoon representation, with amino residues 1-161 colored in grey (overlapping the X-RAY structure of *Aspergillus nidulans* HapX - PDB DOI: 7AW7; https://doi.org/10.2210/pdb7aw7/pdb) (5) and residues 162-491 colored white, except for highlighted regions: CRR-A (orange), CRR-B (green), CRR-C (blue), CRR-D (magenta), conserved C-terminal region 1 (CCTR1; yellow) and CCTR2 (cyan). The zoom in shows a defined structure comprising mainly alpha helices of CCTR1, CCTR2 and CRR-A (model confidence for these regions ranges from Confident to Very High; visible in https://alphafold.ebi.ac.uk/entry/Q4WER3) (3,4). **(B) The conservation analysis of the HapX structure shows high levels of evolutionary conservation of the structure comprising CCTR1, CCTR2 and CRR-A.** Conservation levels were calculated using the ConSurf server (https://consurf.tau.ac.il) (6–11) with residues color-coded based on their evolutionary conservation level (cyan: lowest; white: middle; maroon: highest conservation; see figure for full color scale). Visualization was done using PyMOL Molecular Graphics System, Version 3.0.3, Schrödinger, LLC (12).

SUPPLEMENTARY TABLES

Supplementary Table S1: Transcriptome data of comparative analysis between strains *hapX* and *hapX*$^{A/B/C/D}$.

Supplementary Table S1A – DESeq2 normalized transcriptome dataset

Supplementary Table S1B – Selected differentially expressed genes in *hapX*$^{A/B/C/D}$ compared to *hapX* in +Fe

Supplementary Table S1C – Functional annotation of significantly downregulated genes using FungiFun2

Supplementary Table S2: Strains used in this study.

strain	genotype	reference
AfS77 (wt)	*ATCC46645, ΔakuA::loxP*	(13)
ΔhapX	*AfS77, ΔhapX::ptrA*	(14)
pxylP:hapX	*AfS77, 5'hapX::hph, pxylP:hapX*	this study
pxylP:hapX$^{A/B/C/D}$	*AfS77, 5'hapX::hph, pxylP:hapX*$^{A/B/C/D}$	this study
pxylP:hapX$^{A/B/C}$	*AfS77, 5'hapX::hph, pxylP:hapX*$^{A/B/C}$	this study
pxylP:hapX$^{B/C/D}$	*AfS77, 5'hapX::hph, pxylP:hapX*$^{B/C/D}$	this study
pxylP:hapX$^{A/B/D}$	*AfS77, 5'hapX::hph, pxylP:hapX*$^{A/B/D}$	this study
pxylP:hapX$^{A/C/D}$	*AfS77, 5'hapX::hph, pxylP:hapX*$^{A/C/D}$	this study
pxylP:hapX$^{B/C}$	*AfS77, 5'hapX::hph, pxylP:hapX*$^{B/C}$	this study
pxylP:hapX$^{A/B}$	*AfS77, 5'hapX::hph, pxylP:hapX*$^{A/B}$	this study
pxylP:hapX^{C}	*AfS77, 5'hapX::hph, pxylP:hapX*^{C}	this study
pxylP:hapX^{B}	*AfS77, 5'hapX::hph, pxylP:hapX*^{B}	this study
pxylP:hapX^{464}	*AfS77, 5'hapX::hph, pxylP:hapX*^{464}	this study
pxylP:hapX$^{A/B/C/D;464}$	*AfS77, 5'hapX::hph, pxylP:hapX*$^{A/B/C/D;464}$	this study
cccA^{OE}*; pxylP:hapX*	*AfS77, 5'hapX::hph, pxylP:hapX; 5'fcyB::gpdA:cccA*	this study
cccA^{OE}*; pxylP:hapX*$^{A/B/C/D}$	*AfS77, 5'hapX::hph, pxylP:hapX*$^{A/B/C/D}$*; 5'fcyB::gpdA:cccA*	this study
pxylP:Venus	*AfS77, 5'fcyB::pxylP:Venus*	this study
pxylP:Venus$^{C\text{-}termHapX}$	*AfS77, 5'fcyB::pxylP:Venus*$^{C\text{-}termHapX}$	this study

Supplementary Table S3: Summary of generated plasmids in this study and the encoded *hapX* alleles. Plasmids were either generated applying the NEBuilder or the site-directed mutagenesis (SDM) approach.

plasmid	encoded gene / *hapX* allele	construction method
pMMHL79	*hapX*^{B}	SDM
pSO01	*hapX*$^{A/B}$	SDM
pSO02	*hapX*$^{A/B/C}$	SDM
pSO03	*hapX*$^{A/B/C/D}$	SDM
pSO04	*hapX*$^{A/C/D}$	SDM
pSO05	*hapX*$^{B/C/D}$	SDM
pSO06	*hapX*$^{A/B/D}$	SDM
pSO07	*hapX*$^{A/B/C/D;464}$	SDM
pSO08	*hapX*$^{B/C}$	SDM
pSO09	*hapX*^{464}	SDM
pSO11	*hapX*	NEBuilder
pSO28	*hapX*^{C}	SDM
pSO51	*cccA*	NEBuilder
pSO52	*Venus:hapX*	NEBuilder
pSO53	*Venus*	SDM
pSO54	*Venus*$^{C\text{-}termHapX}$	SDM

Supplementary Table S4: Primers used in this study. Small letters indicate primer overhangs for plasmid construction or introduction of mutations via site-directed mutagenesis (SDM). PC, plasmid construction; TCA, transformation cassette amplification; SB, Southern blot probe; SC, sequencing.

primer	sequence [5' - 3']	used for
pJet1.2 backbone_fwd	ATCTTTCTAGAAGATCTCCTAC	**PC**<br>**➔ pSO11** [*hapX*]
pJet1.2 backbone_rev	ATCTTGCTGAAAAACTCG	**PC**<br>**➔ pSO11** [*hapX*]
5'hapX flank_fwd	ctcgagtttttcagcaagatAGCGACTATAGCCGGATG	**PC**<br>**➔ pSO11** [*hapX*]
5'hapX flank_rev	taaatggtacGATTACGGATGATGAGAC	**PC**<br>**➔ pSO11** [*hapX*]
hph-xylP cassette_fwd	atccgtaatcGTACCATTTAATTCTATTTGTGTTTG	**PC**<br>**➔ pSO11** [*hapX*]
hph-xylP cassette_rev	gtgtagacatGGTTGGTTCTTCGAGTCG	**PC**<br>**➔ pSO11** [*hapX*]
hapX-3'flank_fwd	agaaccaaccATGTCTACACCTTCAATAGC	**PC**<br>**➔ pSO11** [*hapX*]
hapX-3'flank_rev	aggagatcttctagaaagatCCTTGGGTCTTGAAGCTTG	**PC**<br>**➔ pSO11** [*hapX*]
oAfHapX-B1.f	CGTGGATCCCgccGGCTTCTGTTC	**PC – SDM of CRR-B in pSO11**<br>**➔ pMMHL-79** [*hapX*^{B}]
oAfHapX-B1.r	GCAGGAGACGAAACG	**PC – SDM of CRR-B in pSO11**<br>**➔ pMMHL-79** [*hapX*^{B}]
oAfHapX-A2.f	CTGCAATGATgcaTCCACATCGCATTG	**PC – SDM of CRR-A in pMMHL79**<br>**➔ pSO01** [*hapX*$^{A/B}$]
oAfHapX-A2.r	CCTAACGGTACCTCC	**PC – SDM of CRR-A in pMMHL79**<br>**➔ pSO01** [*hapX*$^{A/B}$]
oAfHapX-C3.f	TGCGCGCAGgcgCTTGCAGATCCG	**PC – SDM of CRR-C in pSO01 & pSO11**<br>**➔ pSO02** [*hapX*$^{A/B/C}$] **& pSO28** [*hapX*^{C}]
oAfHapX-C3.r	TGTGCCCGGCCCGTTGGC	**PC – SDM of CRR-C in pSO01 & pSO11**<br>**➔ pSO02** [*hapX*$^{A/B/C}$] **& pSO28** [*hapX*^{C}]
oAfHapX-D2.f	GTCGGGATGCgcaGGAGGTAAAGGCGC	**PC – SDM of CRR-D in pSO02**<br>**➔ pSO03** [*hapX*$^{A/B/C/D}$]
oAfHapX-D2.r	GGGGCAGCGCTAGGG	**PC – SDM of CRR-D in pSO02**<br>**➔ pSO03** [*hapX*$^{A/B/C/D}$]
oAfhapX-SO3	CGTGGATCCCtgtgggTTCTGTTCGGATG	**PC – reverse SDM of CRR-B in pSO03**<br>**➔ pSO04** [*hapX*$^{A/C/D}$]
oAfhapX-SO4	GCAGGAGACGAAACGGAC	**PC – reverse SDM of CRR-B in pSO03**<br>**➔ pSO04** [*hapX*$^{A/C/D}$]
oAfhapX-SO1	CTGCAATGATtgcTCCACATCGCATTGC	**PC – reverse SDM of CRR-A in pSO03 & pSO02**<br>**➔ pSO05** [*hapX*$^{B/C/D}$] **& pSO08** [*hapX*$^{B/C}$]
oAfhapX-SO2	CCTAACGGTACCTCCTC	**PC – reverse SDM of CRR-A in pSO03 & pSO02**<br>**➔ pSO05** [*hapX*$^{B/C/D}$] **& pSO08** [*hapX*$^{B/C}$]
oAfhapX-SO5	AgtgtCTTGCAGATCCGCGGAGGAC	**PC – reverse SDM of CRR-C in pSO03**<br>**➔ pSO06** [*hapX*$^{A/B/D}$]
oAfhapX-SO6	GCGCGCATGTGCCCGGCCCG	**PC – reverse SDM of CRR-C in pSO03**<br>**➔ pSO06** [*hapX*$^{A/B/D}$]
oAfhapX-SO7	TGATTTATCGCATCTCTGCTTG	**PC – SDM (C-terminal truncation) in pSO11 & pSO03**<br>**➔ pSO09** [*hapX*^{464}] **& pSO07** [*hapX*$^{A/B/C/D;464}$]
oAfhapX-SO8	CCCACGATCGGTTAAGGG	**PC – SDM (C-terminal truncation) in pSO11 & pSO03**<br>**➔ pSO09** [*hapX*^{464}] **& pSO07** [*hapX*$^{A/B/C/D;464}$]
fcyB-bb_trpC_fwd	CCATGGCAGCAGTGATTTC	**PC**<br>**➔ pSO52** [*Venus:hapX*]
fcyB-bb_xylP_rev	GGTTGGTTCTTCGAGTCG	**PC**<br>**➔ pSO52** [*Venus:hapX*]
fcyB-bb_rev	AAACAGCTATGACCATGATTAC	**PC**<br>**➔ pSO51** [*cccA*^{OE}]
gpdA promoter_fwd	aatcatggtcatagctgtttGTACCATTTAATTCTATTTGTGTTTG	**PC**<br>**➔ pSO51** [*cccA*^{OE}]

gpdA promoter_rev	aaagcgccatGGGAAAAGAAAGAGAAAAGAAAAG	**PC**<br>➔ **pSO51** [$cccA^{OE}$]
cccA_fwd	ttcttttcccATGGCGCTTTTATTTCTTAAAC	**PC**<br>➔ **pSO51** [$cccA^{OE}$]
cccA_rev	tgaaatcactgctgccatggTCAAGCAGAACCATGGTC	**PC**<br>➔ **pSO51** [$cccA^{OE}$]
Venus_fwd	atcgactcgaagaaccaaccATGGTCAGCAAGGGCGAG	**PC**<br>➔ **pSO52** [*Venus:hapX*]
Venus_linker-seq_rev	gtgtagacatGGTTACGGATGACTTGTACAGCTCGTCCATG	**PC**<br>➔ **pSO52** [*Venus:hapX*]
linker-seq_hapX_fwd	atccgtaaccATGTCTACACCTTCAATAGC	**PC**<br>➔ **pSO52** [*Venus:hapX*]
hapX_rev	tgaaatcactgctgccatggTCATTTGTCGGCAAACCG	**PC**<br>➔ **pSO52** [*Venus:hapX*]
pSO53_fwd	TGACCATGGCAGCAGTGATTTC	**PC – SDM (truncation) in pSO52**<br>➔ **pSO53** [*Venus*]
pSO53_rev	CTTGTACAGCTCGTCCATGC	**PC – SDM (truncation) in pSO52**<br>➔ **pSO53** [*Venus*]
pSO54_fwd	GTCCCAAGGGCCGCTTTG	**PC – SDM (truncation) in pSO52**<br>➔ **pSO54** [$Venus^{27aaC\text{-}termHapX}$]
pSO54_rev	GGTTACGGATGACTTGTACAGCTC	**PC – SDM (truncation) in pSO52**<br>➔ **pSO54** [$Venus^{27aaC\text{-}termHapX}$]
oAfhapX-seq1	TCGGTGGAAAGAAGTGCC	SC
oAfhapX-seq2	CGAGTCCGTTTGGGTATC	SC
oAfhapX-S3	TTTGTCGGCAAACCGTCG	SC
oAfhapX-1	AGCGACTATAGCCGGATG	TCA, SB
oAfhapX-2	CCTTGGGTCTTGAAGCTTGCG	TCA
oAfhapX-4	ATCAGAGCTGGAGAGGCA	SB
oAfhapX-xylP.seq	GTATAAGTATCGCCTCCATC	SC
*5'fcyB*_fwd	CAGAGAATTGCCAAGCTGGT	SB
*5'fcyB*_rev	TAGTTCTGTTACCGAGCCGGCCTGAGTCAATCCCCACCAC	SB

Supplementary Table S5: Primers used for generation of DIG-labelled Northern blot probes.

primer	sequence [5' - 3']	targeted gene	short description
oAfhapX-seq1	TCGGTGGAAAGAAGTGCC	*hapX*<br>Afu5g03920	iron regulatory transcription factor
oAfhapX-S3	TTTGTCGGCAAACCGTCG		
oAfmirB1	AAGCCGAGAAAAAGGGGG	*mirB*<br>Afu3g03640	TAFC transporter
oAfmirB2	AACCCAGATGAAGCCCAG		
oAfsreA5	CTCAGTACGATCGCTTCC	*sreA*<br>Afu5g11260	iron regulatory transcription factor
oAfsreA6	GTTGGACGAGTAGGTAGC		
oAfhemA-f	CAAAGGCAAGACTCCACG	*hemA*<br>Afu5g06270	heme biosynthesis
oAfhemA-r	GGCATCACCAACCAGAAG		
Venus_probe_fwd	GACGTAAACGGCCACAAGTT	*Venus*	yellow-fluorescent protein, Venus
Venus_probe_rev	GAACTCCAGCAGGACCATGT		

SUPPLEMENTARY REFERENCES

1. Priebe,S., Kreisel,C., Horn,F., Guthke,R. and Linde,J. (2015) FungiFun2: a comprehensive online resource for systematic analysis of gene lists from fungal species. *Bioinformatics*, **31**, 445–446.

2. Sayers,E.W., Agarwala,R., Bolton,E.E., Brister,J.R., Canese,K., Clark,K., Connor,R., Fiorini,N., Funk,K., Hefferon,T., *et al.* (2019) Database resources of the National Center for Biotechnology Information. *Nucleic Acids Research*, **47**, D23–D28.

3. Jumper,J., Evans,R., Pritzel,A., Green,T., Figurnov,M., Ronneberger,O., Tunyasuvunakool,K., Bates,R., Žídek,A., Potapenko,A., *et al.* (2021) Highly accurate protein structure prediction with AlphaFold. *Nature*, **596**, 583–589.

4. Varadi,M., Anyango,S., Deshpande,M., Nair,S., Natassia,C., Yordanova,G., Yuan,D., Stroe,O., Wood,G., Laydon,A., *et al.* (2022) AlphaFold Protein Structure Database: massively expanding the structural coverage of protein-sequence space with high-accuracy models. *Nucleic Acids Research*, **50**, D439–D444.

5. Huber,E.M., Hortschansky,P., Scheven,M.T., Misslinger,M., Haas,H., Brakhage,A.A. and Groll,M. (2022) Structural insights into cooperative DNA recognition by the CCAAT-binding complex and its bZIP transcription factor HapX. *Structure*, **30**, 934-946.e4.

6. Yariv,B., Yariv,E., Kessel,A., Masrati,G., Chorin,A.B., Martz,E., Mayrose,I., Pupko,T. and Ben-Tal,N. (2023) Using evolutionary data to make sense of macromolecules with a “face-lifted” ConSurf. *Protein Science*, **32**, e4582.

7. Ashkenazy,H., Abadi,S., Martz,E., Chay,O., Mayrose,I., Pupko,T. and Ben-Tal,N. (2016) ConSurf 2016: an improved methodology to estimate and visualize evolutionary conservation in macromolecules. *Nucleic Acids Research*, **44**, W344–W350.

8. Celniker,G., Nimrod,G., Ashkenazy,H., Glaser,F., Martz,E., Mayrose,I., Pupko,T. and Ben-Tal,N. (2013) ConSurf: Using Evolutionary Data to Raise Testable Hypotheses about Protein Function. *Israel Journal of Chemistry*, **53**, 199–206.

9. Ashkenazy,H., Erez,E., Martz,E., Pupko,T. and Ben-Tal,N. (2010) ConSurf 2010: calculating evolutionary conservation in sequence and structure of proteins and nucleic acids. *Nucleic Acids Research*, **38**, W529–W533.

10. Landau,M., Mayrose,I., Rosenberg,Y., Glaser,F., Martz,E., Pupko,T. and Ben-Tal,N. (2005) ConSurf 2005: the projection of evolutionary conservation scores of residues on protein structures. *Nucleic Acids Research*, **33**, W299–W302.

11. Glaser,F., Pupko,T., Paz,I., Bell,R.E., Bechor-Shental,D., Martz,E. and Ben-Tal,N. (2003) ConSurf: Identification of Functional Regions in Proteins by Surface-Mapping of Phylogenetic Information. *Bioinformatics*, **19**, 163–164.

12. PyMOL Molecular Graphics System, Version 3.0.3, Schrödinger, LLC.

13. Hartmann,T., Dümig,M., Jaber,B.M., Szewczyk,E., Olbermann,P., Morschhäuser,J. and Krappmann,S. (2010) Validation of a self-excising marker in the human pathogen *Aspergillus fumigatus* by employing the beta-rec/six site-specific recombination system. *Appl Environ Microbiol*, **76**, 6313–6317.

14. Gsaller,F., Hortschansky,P., Beattie,S.R., Klammer,V., Tuppatsch,K., Lechner,B.E., Rietzschel,N., Werner,E.R., Vogan,A.A., Chung,D., *et al.* (2014) The Janus transcription factor HapX controls fungal adaptation to both iron starvation and iron excess. *EMBO J*, **33**, 2261–2276.
